# Determinants and health outcomes of trajectories of social mobility in Australia

**DOI:** 10.1016/j.ssmph.2023.101336

**Published:** 2023-01-05

**Authors:** Mithilesh Dronavalli, Andrew Page, Sandro Sperandei, Gabriela Uribe, Carmen Huckel Schneider, John Eastwood

**Affiliations:** a Translational Health Research Institute, Western Sydney University, Australia; b Menzies Centre for Health Policy and Economics, Sydney School of Public Health, University of Sydney, Sydney, Australia; c Sydney Local Health District, Sydney, Australia

## Abstract

**Objectives:**

To investigate trajectories in socio-economic position (SEP) and the onset of a range of physical and mental health outcomes and commencement of treatment.

**Methods:**

The Household Income and Labour Dynamics Australia (HILDA) study, a nationally representative prospective cohort study over the period 2001 to 2020 was used to define trajectories of SEP. Trajectories of low, low-middle, upper-middle and high SEP and decreasing (low-middle to upper-middle SEP) or increasing (upper-middle to lower-middle SEP) SEP were identified using k-longitudinal means. Cox-regression was used to assess SEP trajectories and physical (arthritis or osteoporosis, any cancer, asthma, chronic bronchitis or emphysema, Type 1 diabetes, Type 2 diabetes, hypertension or high blood pressure, and coronary heart disease), and mental health (depression or anxiety) outcomes, and treatment commencement. Predictors of SEP trajectories were also investigated using multinomial logistic regression and random forests.

**Results:**

Decreasing SEP had a higher relative risk of new onset illness than increasing SEP for all health outcomes. Increasing SEP had relative risk estimates that were more consistent with upper-middle income groups and decreasing SEP had a relative risk consistent with lower-middle income groups. In contrast, there was no socio-economic gradient in treatment commencement for physical health outcomes, or depression or anxiety, with the exception of arthritis or osteoporosis.

**Conclusion:**

Decreasing SEP was associated with poor health outcomes, and increasing SEP with better health outcomes. A range of socio-demographic and psychosocial determinants of SEP trajectories were identified to inform policy responses that could modify trajectories of health inequalities in the Australian context.

## Highlights

**What is already known on this topic?**

• The determinants and effect of change in socio-economic position (SEP) over the life-course is largely unclear.

**What this study adds?**

• Decreasing social mobility had a higher relative risk of new onset serious illness than increasing social mobility for all health outcomes. Treatment commencement did not follow a social gradient and was not affected by a change in SEP.
• Modifiable factors of SEP trajectories over the life-course, include education, unemployment, and housing, alcohol consumption, loneliness and social connection.

**How this study might affect research, practice or policy?**

• Interventions to facilitate upward social mobility may include early childhood education, housing affordability, education and training, structured employment support, income re-distribution, and health service access.

## 1 Introduction

Socio-economic position (SEP) is a major determinant of poor health and social outcomes. SEP is determined by socio-economic, cultural and political context, and affects health via material, psychosocial and biobehavioural factors (World Health, 2010), and can have effects over the life course through latent effects, specific pathways or trajectories, accumulation of effects, or via social mobility (Ben-Shlomo & Kuh, 2002; Lynch & Smith, 2005; Niedzwiedz et al., 2012; Pollitt et al., 2005). These outcomes often also follow a social gradient, where there are poorer health outcomes among those of lower (compared to higher) SEP, and these improve as affluence increases There is clear and consistent evidence of a social gradient of association between SEP and a range of outcomes including physical health (Marmot, 2005), mental health and substance use (de Oliveira et al., 2022), crime (Ljujic et al., 2017), COVID-19 hospitalisations and mortality (Eva, 2022) and educational attainment. Reliable statistical evidence for the effect of SEP on educational achievement, dates as far back as 1967 (Sewell VPS, 1967).

Social mobility relates to inter- and intra-generational changes in SEP, based on measures of income, occupational status, and/or educational achievement (Patricia & Rachel, 2020). Recent evidence shows that downward social mobility is associated with a range of health and behavioural outcomes, including depression and other mental health outcomes (Euteneuer & Schafer, 2018; Melchior et al., 2018),hypertension (Lopes et al., 2021) and, acute coronary syndrome. (Burazeri et al., 2008), lower leisure time physical activity (Elhakeem et al., 2016), and increased tobacco consumption (Motta et al., 2015). Improving social mobility for discriminated groups in a community can lead to lower cause-specific cancer mortality for the whole community (Poulson et al., 2022).There are also differential impacts of social mobility on health among migrant groups (associated with migration to lower relative SEP settings and under-employment) (Das-Munshi et al., 2012) and different points across the life course (Fu et al., 2022; Niedzwiedz et al., 2012) Much of this evidence arises from population cohort studies including the French nationally representative CONSTANCES study of 67,057 individuals (Melchior et al., 2018) or the European Social Survey of 52,773 individuals (Gugushvili et al., 2019) or the ELSA-Brasil study of 8754 participants (Lopes et al., 2021). These are high quality prospective cohorts with follow-up over many years.

In the Australian context, there has been extensive previous research investigating health inequalities based on socio-economic position (Mech et al., 2016; Shepherd et al., 2012; Williams et al., 2013), including for a range of chronic disease outcomes, health service access, and trends in socio-economic differentials over time. However, there has been limited investigation of how trajectories in social mobility (that is, changes in SEP) over the life-course affects health outcomes and health service access, and whether these trajectories are specific to particular health outcomes or are consistent across health outcomes.

Accordingly, this research aims to investigate the association between SEP and the impact of upward and downward social mobility on the onset of a range of (i) physical health outcomes (obesity, cardiovascular disease, diabetes, respiratory disease, and cancer), (ii) mental health outcomes (psychological distress, depression or anxiety) and (iii) associated treatment commencement, based on a large representative prospective cohort study of Australia (Roger Wilkins and BothaSarah, 2021). An additional aim was to identify socio-demographic and psychosocial determinants associated with changes in SEP (social mobility). Potential determinants included a wide range of time-varying variables, for example educational achievement, unemployment, marital status, alcohol consumption, body-mass index (BMI), Kessler 10 distress score, among others. This research can inform policy responses in both preventing downward social mobility as a distal factor associated with poor health, and in ameliorating the impact of falling or low SEP on new onset serious chronic diseases.

## 2 Methods

### 2.1 Study setting

This study uses a prospective cohort study design based on the nation-wide Household, Income, and Labour Dynamics Australia (HILDA) survey designed by the Melbourne Institute of Applied Economic and Social Research at The University of Melbourne and funded by the Australian government through the Department Of Social Services. The HILDA cohort is a representative longitudinal cohort of people residing in Australia for the period 2001 to 2020 (N = 34,573 people ever followed up and 22,932 people at last follow-up). The HILDA study data consists of self-reported questionnaires on domains including family background, health, education, income and wealth, housing, relationships, and employment over 20 waves of follow-up. A detailed description of this cohort is presented elsewhere (Roger Wilkins and BothaSarah, 2021). The data dictionary, summary frequencies and response rates for each variable and across each wave from wave 1 to 20, is also documented elsewhere (Melbourne Institute, 2022). Participants enter and leave the cohort, with an average age of 48.5 years.

### 2.2 SEP and social mobility

**Fig. 1:**
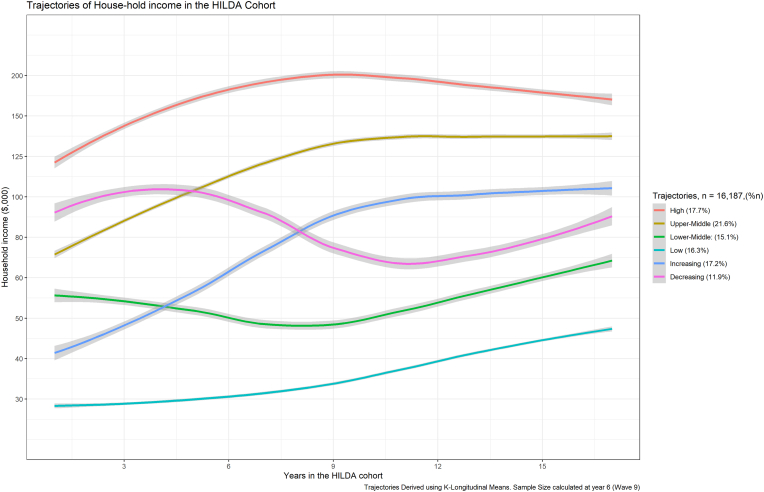
Trajectories of SEP over time, Household Income and Labour Dynamics (HILDA) study, Australia, 2001–2020. Caption: Trajectories derived using K-Longitudinal means. Cross-section data reported in Table 1, Table 2 corresponds to year 6 (Wave 9).

The main exposure of interest was SEP, and how SEP changed over the course of follow-up in the HILDA cohort. Categories of social mobility were based on household income (adjusted for inflation over time), collected for seventeen waves of follow-up, and derived empirically using the K-means longitudinal clustering approach (Genolini et al., 2015). This approach grouped similar trajectories of people in the cohort into clusters based on the principles of K-means. Six clusters of similar proportions of the cohort (12%–22%) were generated automatically (not *a priori*) based on un-supervised learning from patterns of trajectories in household income using the R-package kml. For each cluster 60%–80% of participants had house-hold income recorded for the full 17 years of follow-up (Genolini et al., 2015). Five or six clusters were recommended to optimise various information criteria such as the Calinkski-Harbatz coefficient, AIC, BIC, and Ray-Turi index. Six clusters were identified as interpretable clusters of SEP in this study (Fig. 1). The K-means longitudinal method identified 4 clearly delineated clusters across a range of stable SEPs, and 2 clusters of rising or falling SEP, over time (Table 1, Table 2, Fig. 1). Kml requires that there is no missing data for anyone in the analysis, so household incomes were carried forward for a complete case analysis without extrapolation. Imputation of data from the HILDA cohort has already been carried out by the data custodians at the Melbourne Institute (Breunig, 2022). There were four stable groups (that is, where participants did not change their SEP) who were defined as ‘High Income’ ([18%, N = 2780]), ‘Upper Middle Income’([22%, N = 3593]), ‘Lower Middle Income’([15%, N = 2451]) and ‘Low Income’([16%, N = 2830]). There were also two groups where participants' SEP changed over time, defined as changes from higher to lower SEP (that is, ‘decreasing’ social mobility, N = 2003, or 12% of the cohort), and from lower to higher SEP (that is, ‘increasing’ social mobility, N = 2530 or 17% of the cohort).

**Table 1:** Descriptive statistics for social mobility trajectories (increasing and decreasing SEP), Household Income and Labour Dynamics (HILDA) study, Australia, 2001–2020.

Variable Name	Increasing SEP Mean(SD) or Prop. (%)	N	Decreasing SEP Mean(SD) or Prop. (%)	N	Missing Mean(SD) or Prop. (%)	N	Total Mean(SD) or Prop. (%)	N
Age at Wave 9	40.3 (13.8)	2530	48.6 (12.4)	2003	40.8 (19.8)	1442	45.8 (15.9)	17629
K10 score	15.7 (6.0	1669	15.4 (5.8)	1494	16.9 (6.9)	679	15.7 (6.2)	12129
BMI	26.3 (5.4)	1704	26.6 (5.4)	1524	26.1 (6.0)	654	26.5 (5.4)	12294
Time Unemployed Last Financial Year	5%	1826	4%	1602	8%	947	5%	13300
Female	50%	2530	53%	2003	48%	1442	0%	17629
Male	50%	2530	47%	2003	52%	1442	50%	17629
*Accessiblity/Remoteness Index of Australia*								
Live in a City	55%	2530	61%	2003	58%	1442	61%	17632
Inner Regional Australia	29%	2530	27%	2003	25%	1442	25%	17632
Outer Regional Australia	13%	2530	11%	2003	14%	1442	12%	17632
Remote Australia	3%	2530	2%	2003	2%	1442	2%	17632
Very Remote Australia	0%	2530	0%	2003	1%	1442	0%	17632
*Highest Educational Qualification*								
Masters or PhD	2%	1826	3%	1602	1%	948	4%	13301
Graduate Diploma or Grad Certificate	3%	1826	5%	1602	3%	948	5%	13301
Bachelor or Honours	11%	1826	10%	1602	11%	948	12%	13301
Advanced Diploma	8%	1826	9%	1602	7%	948	8%	13301
Cert III or IV	24%	1826	21%	1602	19%	948	20%	13301
Year 12	22%	1826	17%	1602	17%	948	16%	13301
Year 11 and Below	30%	1826	34%	1602	41%	948	35%	13301
Undetermined	0%	1826	0%	1602	0%	948	0%	13301
*Alcohol Consumption*								
I have never drunk alcohol	14%	2530	8%	2003	9%	1442	8%	17632
I no longer drink	10%	2530	4%	2003	4%	1442	5%	17632
I drink alcohol everyday	8%	2530	5%	2003	6%	1442	6%	17632
I drink alcohol 5 or 6 days per week	6%	2530	5%	2003	8%	1442	6%	17632
I drink alcohol 3 or 4 days per week	7%	2530	9%	2003	11%	1442	10%	17632
I drink alcohol 1 or 2 days per week	10%	2530	15%	2003	15%	1442	15%	17632
I drink alcohol 2 or 3 days per week	6%	2530	10%	2003	11%	1442	9%	17632
I drink only rarely	24%	2530	17%	2003	17%	1442	17%	17632
*Marital Status*								
Married	40%	1826	53%	1602	27%	947	0%	13300
Separated but not divorced	3%	1826	3%	1602	2%	947	3%	13300
Divorced	8%	1826	9%	1602	9%	947	9%	13300
Widowed	2%	1826	2%	1602	9%	947	5%	13300
Never married but in a relationship	16%	1826	7%	1602	19%	947	10%	13300
Never married and not with someone	32%	1826	26%	1602	34%	947	25%	13300
Speaks Language Other Than English	11%	1826	8%	1602	9%	946	10%	13295
*Serious Health Outcomes*								
Any Serious Illness	34%	2530	41%	2003	44%	1442	42%	17632
Arthritis/Osteoporosis	31%	2530	33%	2003	32%	1442	34%	17632
Cancer	7%	2530	7%	2003	5%	1442	7%	17632
Chronic Bronchitis/Emphysema	3%	2530	2%	2003	3%	1442	4%	17632
Asthma	21%	2530	15%	2003	8%	1442	9%	17632
Type 1 Diabetes	2%	2530	2%	2003	3%	1442	2%	17632
Type 2 Diabetes	8%	2530	8%	2003	10%	1442	9%	17632
Depression or Anxiety	32%	2530	30%	2003	33%	1442	31%	17632
Heart Disease	7%	2530	8%	2003	8%	1442	9%	17632
High Blood Pressure	30%	2530	34%	2003	28%	1442	33%	17632
*Treatment for Serious Health Outcomes*								
Arthritis or Osteoporosis	31%	2530	29%	2003	28%	1442	31%	17632
Asthma	22%	2530	19%	2003	27%	1442	22%	17632
Cancer	4%	2530	4%	2003	6%	1442	5%	17632
Chronic Bronchitis/Emphysema	5%	2530	4%	2003	4%	1442	5%	17632
Type 1 Diabetes	2%	2530	3%	2003	4%	1442	3%	17632
Type 2 Diabetes	14%	2530	14%	2003	13%	1442	14%	17632
Depression or Anxiety	38%	2530	35%	2003	41%	1442	35%	17632
Has Seen a Mental Health Professional	10%	1042	9%	1053	9%	604	9%	8679
Heart Disease	14%	180	13%	223	22%	174	19%	2213
High Blood Pressure	45%	180	64%	223	49%	174	59%	2213

**Table 2:** Descriptive statistics for SEP groups (high, upper-middle, lower-middle, low), Household Income and Labour Dynamics (HILDA) study, Australia, 2001–2020.

Variable Name	High SEP Mean(SD) or Prop. (%)	N	Upper-Middle SEP Mean(SD) or Prop. (%)	N	Lower-Middle SEP Mean(SD) or Prop. (%)	N	Low SEP Mean(SD) or Prop. (%)	N
Age at Wave 9	43.2 (9.9)	2780	42.1 (11.2)	3593	46.4 (17.2)	2451	57.9 (19.3)	2830
K10 Score	14.3 (4.8)	1920	14.9 (5.3)	2489	16.5 (6.8)	1678	17.0 (7.5)	2200
BMI	25.9 (4.7)	1956	26.4 (5.1)	2536	26.6 (5.8)	1703	27.3 (5.8)	2217
Time Unemployed Last Financial Year	2%	2031	3%	2651	7%	1812	6%	2431
Female	55%	2780	53%	3593	47%	2451	43%	2830
Male	46%	2780	47%	3593	53%	2451	57%	2830
*Accessiblity/Remoteness Index of Australia*								
Live in a City	80%	2780	65%	3593	54%	2451	48%	2833
Inner Regional Australia	14%	2780	24%	3593	28%	2451	32%	2833
Outer Regional Australia	5%	2780	9%	3593	16%	2451	17%	2833
Remote Australia	1%	2780	2%	3593	1%	2451	2%	2833
Very Remote Australia	0%	2780	1%	3593	0%	2451	0%	2833
*Highest Educational Qualification*								
Masters or PhD	11%	2031	4%	2651	2%	1812	1%	2431
Graduate Diploma or Grad Certificate	12%	2031	6%	2651	2%	1812	2%	2431
Bachelor or Honours	26%	2031	16%	2651	6%	1812	5%	2431
Advanced Diploma	10%	2031	10%	2651	9%	1812	6%	2431
Cert III or IV	13%	2031	22%	2651	21%	1812	19%	2431
Year 12	13%	2031	18%	2651	15%	1812	11%	2431
Year 11 and Below	15%	2031	23%	2651	44%	1812	58%	2431
Undetermined	0%	2031	0%	2651	0%	1812	0%	2431
*Alcohol Consumption*								
I have never drunk alcohol	5%	2780	5%	3593	10%	2451	14%	2833
I no longer drink	2%	2780	4%	3593	5%	2451	10%	2833
I drink alcohol everyday	5%	2780	5%	3593	6%	2451	8%	2833
I drink alcohol 5 or 6 days per week	9%	2780	6%	3593	6%	2451	6%	2833
I drink alcohol 3 or 4 days per week	13%	2780	11%	3593	8%	2451	7%	2833
I drink alcohol 1 or 2 days per week	18%	2780	18%	3593	12%	2451	10%	2833
I drink alcohol 2 or 3 days per week	10%	2780	11%	3593	8%	2451	6%	2833
I drink only rarely	13%	2780	15%	3593	19%	2451	24%	2833
*Marital status*								
Married	63%	2031	55%	2651	42%	1812	37%	2431
Separated but not divorced	2%	2031	2%	2651	5%	1812	4%	2431
Divorced	5%	2031	6%	2651	10%	1812	16%	2431
Widowed	0%	2031	1%	2651	5%	1812	19%	2431
Never married but in a relationship	11%	2031	12%	2651	9%	1812	3%	2431
Never married and not with someone	19%	2031	24%	2651	29%	1812	20%	2431
Speaks Language Other Than English	9%	2031	8%	2651	10%	1811	12%	2428
*Serious health outcome*								
Any Serious Illness	30%	2780	33%	3593	49%	2451	68%	2833
Arthritis/Osteoporosis	32%	2780	31%	3593	34%	2451	45%	2833
Cancer	7%	2780	7%	3593	7%	2451	8%	2833
Chronic Bronchitis/Emphysema	3%	2780	3%	3593	5%	2451	7%	2833
Asthma	12%	2780	19%	3593	16%	2451	15%	2833
Type 1 Diabetes	2%	2780	2%	3593	1%	2451	2%	2833
Type 2 Diabetes	8%	2780	7%	3593	9%	2451	13%	2833
Depression or Anxiety	1%	2530	1%	2003	1%	1442	8%	17632
Other Mental Illness	4%	2780	3%	3593	5%	2451	5%	2833
Any Serious Mental Illness	1%	2780	1%	3593	1%	2451	3%	2833
Serious Heart Disease	8%	2780	7%	3593	9%	2451	13%	2833
High Blood Pressure	33%	2780	30%	3593	32%	2451	44%	2833
Peripheral Vascular Disease/Stroke	4%	2780	4%	3593	6%	2451	8%	2833
CVD/PVD/High Blood Pressure	37.0%	583	32%	827	43%	908	58%	1733
*Treatment for Serious Health Outcomes*								
Arthritis or Osteoporosis	28%	2780	30%	3593	31%	2451	36%	2833
Asthma	22%	2780	23%	3593	24%	2451	22%	2833
Cancer	4%	2780	5%	3593	4%	2451	4%	2833
Chronic Bronchitis/Emphysema	5%	2780	4%	3593	5%	2451	6%	2833
Type 1 Diabetes	3%	2780	3%	3593	2%	2451	3%	2833
Type 2 Diabetes	14%	2780	14%	3593	13%	2451	16%	2833
Depression or Anxiety	34%	2780	34%	3593	36%	2451	31%	2833
Other Mental Illness	3%	118	6%	156	5%	380	5%	982
Has Seen a Mental Health Professional	7%	1411	8%	1680	11%	1132	9%	1757
Any Serious Mental Illness	8%	1426	9%	1698	16%	1181	19%	1878
Heart Disease	19%	118	15%	156	19%	380	22%	982
High Blood Pressure	58%	118	49%	156	59%	380	64%	982
Peripheral Vascular Disease or Stroke	3%	118	1%	156	11%	380	10%	982
CVD/PVD/High Blood Pressure	64%	118	56%	156	67%	380	74%	982

For the derived social mobility groups, decreasing SEP commenced at the upper middle SEP category and decreased to the lower middle SEP category. In contrast the increasing SEP group started at the lower middle SEP category and increased to the upper middle SEP category (Fig. 1).

### 2.3 Outcome variables

All of the mental health, physical health and treatment initiation outcomes were based on questions like: “Have you ever been diagnosed with XXXX serious illness?”

#### 2.3.1 Onset of mental health outcomes

Mental health outcomes included self-reported diagnosis of depression or anxiety, and psychological distress. Psychological distress was measured as a time-varying variable using the Kessler 10 (K10) questionnaire score. The K10 score is measured every alternative wave from Wave 7 and was carried forward between waves. K10 is higher with rising distress and is a marker of allostatic stress over time. The K10 contains ten questions on symptoms of anxiety and depression. Note however, that K10 is not diagnostic of depression or anxiety. Onset of depression or anxiety was based on responses to questions in wave 13 and wave 17 of follow-up among participants who previously did not report the outcome (as described below).

#### 2.3.2 Onset of physical health outcomes

Physical health outcomes included self-reported past clinical diagnoses of arthritis or osteoporosis, any type of cancer, asthma, chronic bronchitis or emphysema, Type 1 diabetes, Type 2 diabetes, hypertension or high blood pressure, and coronary heart disease. BMI was recorded annually from Wave 6. Onset of physical health outcomes was based on responses to questions in wave 13 and wave 17 of follow-up among participants who previously did not report the outcome at Wave 9 (as described below).

#### 2.3.3 Onset of treatments

Additional outcome variables included initiation of treatments associated with the physical and mental health outcomes described above, each as separate variables. Onset of initiation of treatment variables were recorded only in wave 9, wave 13 and wave 17 of follow-up as either ‘yes’ or ‘no’.

### 2.4 Other study factors

A range of other socio-demographic, health and wellbeing factors that were likely associated with the identified social clusters were also identified and included as potential confounders in analyses (a cross-section of the study at wave 9 is presented in Table 1, Table 2). Descriptive data for wave 9 are presented as this was the point at which new onset physical or mental illness at treatment association rates were defined for survival analyses (as described below). Socio-demographic factors included age at when the participant entered the study, gender (‘male’, ‘female’ – gender at entry to the cohort), indigenous status (‘Aboriginal, Torres-Strait Islander’, ‘both Aboriginal and Torres Strait Islander’, ‘neither Aboriginal or Torres Strait Islander’ – measured annually since Wave 1), highest level of educational achievement (‘Masters or PhD’, ‘Graduate Diploma or Grad Certificate’, ‘Bachelor or Honours’, ‘Advanced Diploma’, ‘Cert III or IV’, ‘Year 12’, ‘Year 11 and Below’, ‘Undetermined’ – annual wave 1), marital status (‘married’, ‘separated but not divorced’, ‘divorced’, ‘widowed’, ‘never married but not in a relationship’, ‘never married and not with someone’ – annually since Wave 2), and the percent of time in the previous year spent unemployed (annually since Wave 1). Dwelling type (‘Free Standing House’, ‘Non-private dwelling - nursing homes’, ‘Non-private dwelling - others’, ‘Separate house with attached shop, office, etc’, ‘Semi-detached house with one storey’, ‘Semi-detached house with two or more storeys’, ‘Semi-detached house attached to a shop, office etc’, ‘Flat/unit/apartment in one-storey block’, ‘Flat/unit/apartment in two-storey block’, ‘Flat/unit/apartment in three-storey block’, ‘Flat/unit/apartment in four to nine-storey block’, ‘Flat/unit/apartment in ten or more storey block’, ‘Flat/unit/apartment attached to a house’, ‘Flat/unit/apartment attached to a shop, office etc’, ‘Caravan/Tent/Cabin/Houseboat’ – annually from Wave 2), and the Accessibility/Remoteness Index of Australia (ARIA) (‘Live in a City’, ‘Inner Regional Australia’. ‘Outer Regional Australia’, ‘Remote Australia’, ‘Very Remote Australia’ – annually from Wave 1), Speaks Language Other Than English at home -annually from Wave 2, was also included. Additional health and wellbeing factors included participant responses to questions relating to self-reported physical and emotional problems affecting social functioning (measured on a 5-point Likert scale from 1 to 5 – annually from wave 1), general health and wellbeing (measured on 5-point Likert scale from 1 to 5 – annually from Wave 1), weekly alcohol consumption (‘I have never drunk alcohol’, ‘I no longer drink’, ‘I drink alcohol everyday’, ‘I drink alcohol 5 or 6 days per week’, ‘I drink alcohol 3 or 4 days per week’, ‘I drink alcohol 1 or 2 days per week’, ‘I drink alcohol 2 or 3 days per week’, ‘I drink only rarely’ – annually from Wave 2), and significant life-events in the past year (including birth of a child, victim of physical violence, or incarceration in the household – all annually from Wave 2). Detailed descriptions of these variables can be found in the HILDA data dictionary (Melbourne Institute, 2022).

### 2.5 Data analysis

#### 2.5.1 Health and treatment outcomes

Survival analysis using Cox-Proportional Hazard regression was used to assess the association between SEP trajectories and new onset of each physical health, mental health, and treatment outcome as described above. Participants were selected at Wave 9 if they did not report the described health outcome or treatment. The specific illness or treatment status was measured again at Wave 13 or Wave 17 and these waves indicated the survival outcomes, allowing for new onset health outcomes to be identified.

Confounders were classified as fixed at baseline, or time varying covariates if they changed over time, for each of the outcome variables of interest. Fixed confounders were age at entry to the study, gender, and whether the person spoke a language other than English. Time-varying confounders included percent of time unemployed in the last financial year, urban-rural residence, marital status, highest level of education, indigenous status and alcohol consumption.

Random effects analyses (where the individual person identifier was the random intercept) was used to assess the temporal association between SEP category and BMI (continuous random effects regression) and psychological distress as measured by the discrete K10 score (random effects negative binomial regression) with the same approach to fixed and time-varying confounder adjustment as described above.

#### 2.5.2 Factors associated with SEP and social mobility

Additional analyses also investigated those socio-demographic, health and wellbeing factors associated with SEP and social mobility. A multinomial logistic regression model was developed where SEP clusters were specified as the outcome and age, gender, socio-demographic (dwelling type, highest education qualification, marital status, percent of time unemployed, remoteness of residence (ARIA), English as the language spoken at home, First Nation's Status, life events in the last year, (member of the family jailed, victim of physical violence, birth/adoption of a new child), general health and wellbeing question (1–5), social functioning given health status (1–5), distress score (K10), body mass index were specified as exposures, with individuals defined within each cluster. Decreasing SEP, increasing SEP, high, low-middle, low, were compared to upper-middle SEP as the reference category. Upper-middle SEP was chosen as this income trajectory was the origin of decreasing SEP and destination of the increasing SEP.

An explanation of the equations is given for multinomial logistic regression:

$$\begin{array}{c} In\frac{\mathrm{Pr}\left(Y_{i}=1\right)}{\mathrm{Pr}\left(Y_{i}=K\right)}=\beta_{1}∙X_{i}\\ In\frac{\mathrm{Pr}\left(Y_{i}=2\right)}{\mathrm{Pr}\left(Y_{i}=K\right)}=\beta_{2}∙X_{i}\\ \cdots \cdots \\ In\frac{\mathrm{Pr}\left(Y_{i}=K-1\right)}{\mathrm{Pr}\left(Y_{i}=K\right)}=\beta_{K-1}∙X_{i} \end{array}$$

Pr(*Y*_*i*_ *= K*) is the probability of a participant being in the upper-middle SEP, which is the Kth or sixth cluster. Each other cluster is assigned a value between 1 and *K-1* or one to five. The variables or columns in the dataset are represented by **x**_*i*_. The corresponding **β**_*K-1*_ vector or vector of coefficients exists for each of the other five (*K-1*) clusters where the reference group is the upper-middle SEP cluster. These **β**′s are exponentiated to give a relative risk. In this study this model was extended where each individual's information across all the waves were used as clusters in a multinomial logistic regression random effects model.

Finally, an atheoretical prediction model was also constructed to classify people into categories of SEP or social mobility. The aim of this analysis was to predict those who experienced a decreasing SEP. The cohort was divided into a ‘test’ dataset (20% of the cohort) and a ‘training’ dataset (80% of the cohort). Seven techniques were screened to identify the technique with the lowest cross-over error rate, including random forest (55.3%), multinomial regression (68.2%), regularised multinomial regression (68.2%), k-nearest neighbours (68.9%), neural nets (62.9%), naïve Bayes (78%) and supported vector machines. The random forest technique had the lowest cross-over error rate and is presented in the current study. Also, the random forest technique is most adaptive to models where there is a combination of categorical and continuous predictors and multi-class outcomes, especially in large datasets (Fu et al., 2022). The random forest technique combines a range of decision trees with continuous variables classified at different cut-points. The combination of these decision trees is conducted randomly while optimising desirable model features such as error, strength of individual trees in the random forest and correlation between trees in the random forest (Breiman, 2001).

The random forest technique was optimised in R. Based on the data, an optimal mtry value of 8 was calculated and used for the random forest model. The mtry defines the number of variables randomly selected as candidates at each split. The variables used were ranked in order of the mean decrease in the Gini co-efficient and mean decrease in accuracy when the corresponding variable was removed from the model. The random forest model was evaluated using the measures of sensitivity, specificity, positive predictive value, negative predictive value, prevalence, detection rate, detection prevalence and balanced accuracy.

### 2.6 Missing data

Information on health outcomes were first measured from wave 9 of the HILDA survey. In the HILDA cohort there were 17,632 people enrolled at Wave 9. This system of classification of household income level categories using k-longitudinal means and a minimum of 7 years follow-up classified 16,187 people out of 17,632 (92%).

## 3 Results

### 3.1 SEP and social mobility and new onset chronic disease

**Fig. 2:**
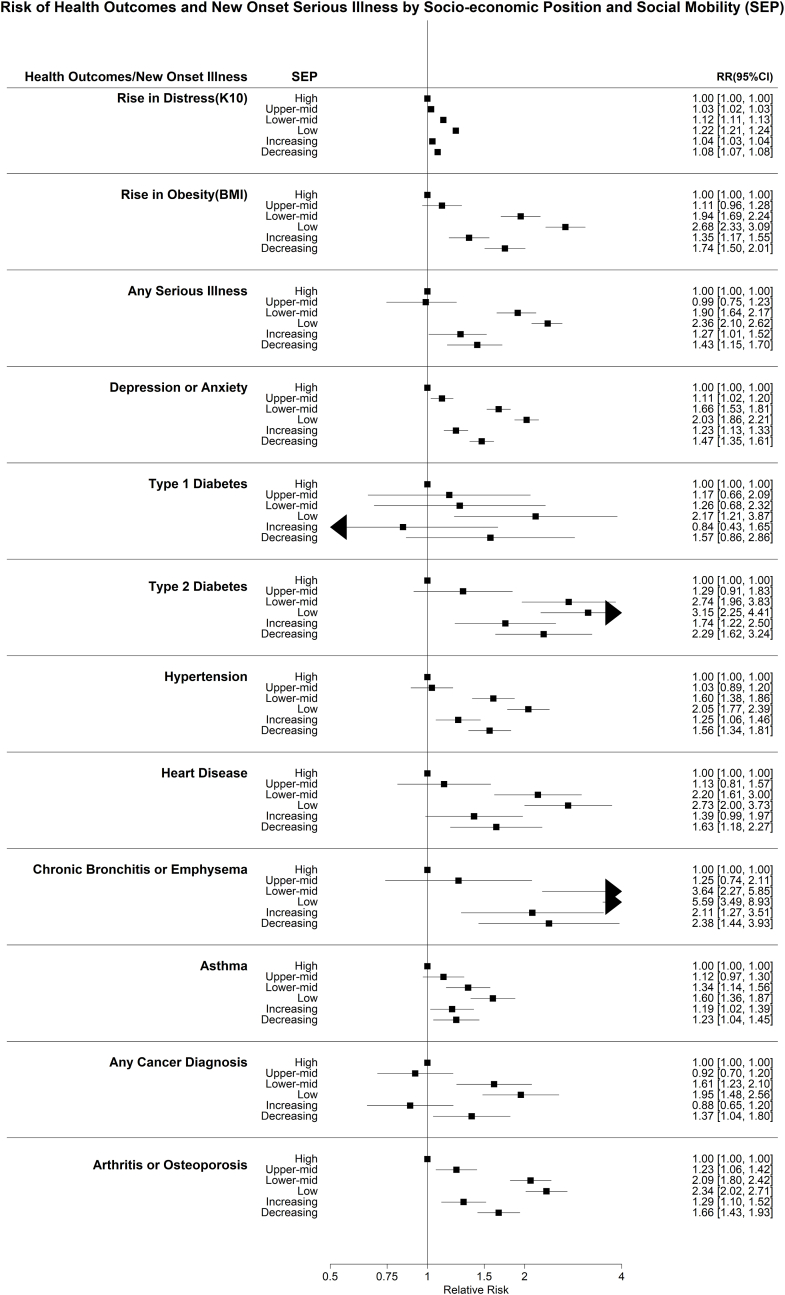
Risk of health outcome and new onset serious illness by trajectory of SEP Household Income and Labour Dynamics (HILDA) study, Australia, 2001–2020. Caption: Relative Risk of new onset physical or mental illness with ‘High’ socio-economic position as the reference group. Figure also includes longitudinal risk of increase in distress (K10) and obesity (BMI) with ‘High’ Socio-economic position as the reference group. Treatment Initiation Rates of physical and mental illnesses by Socio-economic Position. All socio-economic positions including increasing and decreasing socio-economic position are compared to the ‘High’ socio-economic position.

An increasing socio-economic gradient (from highest to lowest SEP) was evident for each of the included health outcomes (Fig. 2), with the highest relative risk evident among those of low SEP compared to high SEP for any serious illness (HR = 2.36, 95%CI 2.10–2.62), cancer (HR = 1.95, 95%CI 1.48–2.56), arthritis or osteoporosis (HR = 2.34, 95%CI 2.02–2.72), bronchitis or emphysema (HR = 5.59, 95%CI 3.49–8.93), asthma (HR = 1.60, 95%CI 1.36–1.87), Type I diabetes (HR = 2.17, 95%CI 1.21–2.87), Type II diabetes (HR = 3.15, 95%CI 2.25–4.41), hypertension (HR = 2.05, 95%CI 1.77–2.39), cardiovascular disease (HR = 2.73, 95%CI 2.00 to 3.73), and depression/anxiety (HR = 2.03, 95%CI: 1.86–2.21).

For all health outcomes, those participants with decreasing social mobility (from higher to low SEP) generally had higher relative risk of illness than those with increasing social mobility, as compared to the high SEP referent category, with the exception of asthma and chronic bronchitis of emphysema (Fig. 2). Participants with decreasing social mobility (from higher to lower SEP) had relative risk estimates that were more consistent with low-middle SEP groups. Participants with increasing SEP (from low-middle to upper-middle SEP) had relative risk estimates that were more consistent with upper-middle income groups (Fig. 2).

**Fig. 3:**
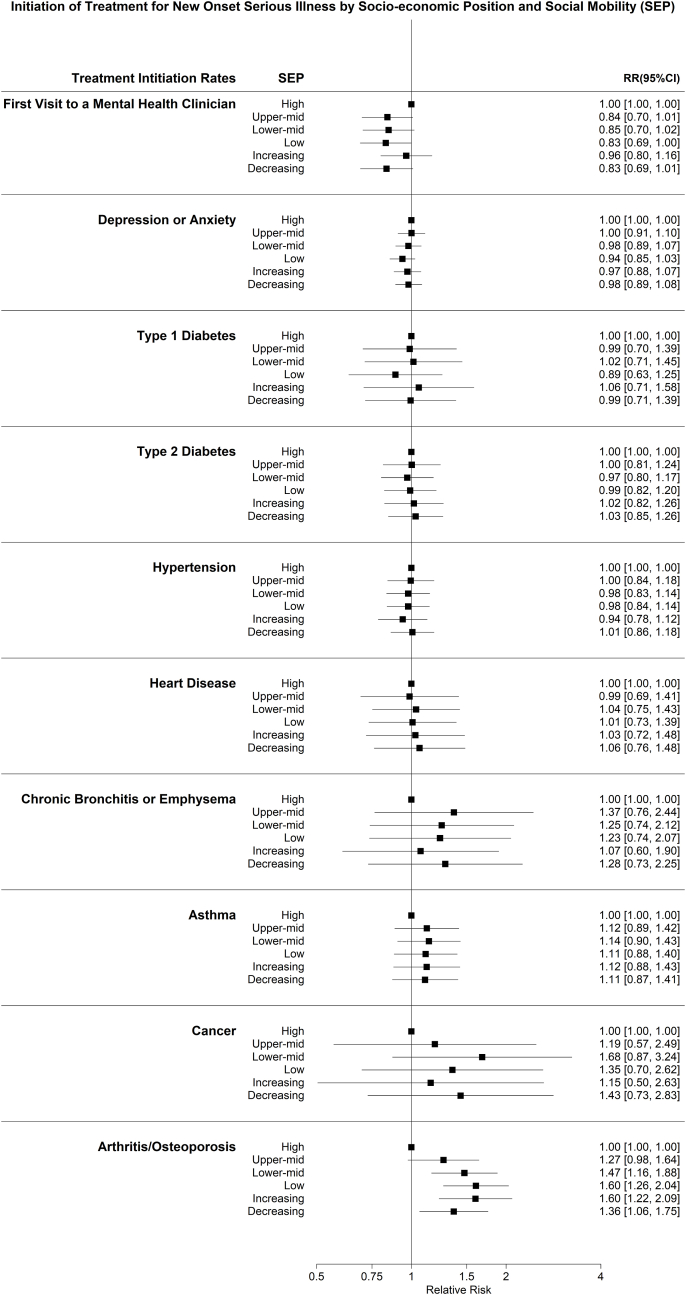
Initiation of treatment for new onset serious illness by trajectory of SEP, Household Income and Labour Dynamics (HILDA) study, Australia, 2001–2020. Caption: Treatment Initiation Rates of physical and mental illnesses by Socio-economic Position. All socio-economic positions including increasing and decreasing socio-economic position are compared to the ‘High’ socio-economic position.

In contrast, there was no evidence of a socio-economic gradient in self-reported treatment commencement for all health outcomes (Fig. 3), with no difference between lower SEP, or between increasing or decreasing SEP groups, compared to the high SEP referent category. The exception was for arthritis or osteoporosis treatment commencement, where those of lower SEP and either decreasing or increasing SEP were more likely to have commenced treatment (Fig. 3). Additionally, those with lower SEP and those of decreasing SEP, were less likely to visit a mental health clinician, compared to the high SEP group (Fig. 3).

### 3.2 Determinants of falling SEP

The results of the full multinomial regression model are provided in Supplementary Materials Table 1. Of particular interest was the comparison between upper-middle SEP and decreasing SEP groups (Table 3). Factors associated with decreasing SEP included older age; being female, general health and wellbeing, social functioning given health, highest level of educational qualifications, not being in a marital relationship, period of unemployment in the last year, remoteness of residence, being a victim of physical violence and not living in a free-standing house. Variables used in the full multinomial regression model were not highly correlated with the highest correlation coefficient at r = −0.61 for K10 (distress) and social functioning given health (not sown).

**Table 3:** Determinants of decreasing SEP, Household Income and Labour Dynamics (HILDA) study, Australia, 2001–2020. N = 15,282 participants. Note: Relative Risk Ratios are for decreasing SEP compared to the Upper-Middle SEP referent group. Decreasing SEP cluster starts at Upper-Middle SEP at beginning of follow-up. Comparisons for all other SEP categories from the multinomial logistic regression are provided in Supplementary Materials.

Study factor	RRR (95%CI)	P-Value
*Age*	1.02 (1.02–1.03)	P < 0.001
*Sex*		
Male	1	
Female	1.16 (1.04–1.30)	0.01
*Self assessed Health and Wellbeing (1–5)*	1.15 (1.09–1.222)	P < 0.001
*Self assessed Social Functioning given health (1–5)*	0.92 (0.88–0.96)	P < 0.001
*Highest Level of Educational Qualification*	1.07 (1.05–1.01)	P < 0.001
*Marital Status*		
Married	1	
Separated but not Divorced	1.99 (1.54–2.56)	P < 0.001
Divorced	1.74 (1.40–2.15)	P < 0.001
Widowed	3.83 (2.46–5.97)	P < 0.001
Never Married but Living with Someone in a relationship	1.26 (1.06–1.48)	0.008
Never married and not living with someone in a relationship	1.57 (1.37–1.81)	P < 0.001
Percent Time Unemployed in last financial year	1.01 (1.01–1.01)	P < 0.001
*K10 score for distress*	1.01 (1.00–1.01)	0.14
*Speaks Language Other Than English*		
No		
Yes	1.29 (0.96–1.74)	0.10
*Alcohol Consumption*		
I have never drunk alcohol	1	
I no longer drink	1.30 (1.03–1.62)	0.02
Yes, I drink alcohol everyday	1.07 (0.82–1.38)	0.63
Yes, I drink alcohol 5 or 6 days per week	0.97 (0.77–1.22)	0.78
Yes, I drink alcohol 3 or 4 days per week	0.86 (0.69–1.06)	0.15
Yes, I drink alcohol 1 or 2 days per week	0.80 (0.65–0.96)	0.02
Yes, I drink alcohol 2 or 3 days per month	0.84 (0.69–1.02)	0.07
Yes, but only rarely	0.94 (0.78–1.13)	0.50
*Accessibility/Remoteness Index of Australia*	1.19 (1.10–1.23)	P < 0.001
*Close Family Member Detained in Jail*		
No		
Yes	1.32 (0.98–1.77)	0.06
*Victim of Physical Violence in past year*		
No		
Yes	1.45 (1.14–1.84)	0.01
*Birth of Child in past year*		
No		
Yes	0.92 (0.79–1.06)	0.24
*Dwelling Type Baseline:*		
Free Standing House	1	
Non-private dwelling - nursing homes	0.67 (0.11–4.17)	0.67
Non-private dwelling - others	11.73 (5.92–23.2)	P < 0.001
Separate house with attached shop, office, etc	1.08 (0.69–1.69)	0.74
Semi-detached house with one storey	1.888 (1.50499–2.35)	P < 0.001
Semi-detached house with two or more storeys	1.51 (1.21–1.89)	P < 0.001
Semi-detached house attached to a shop, office etc	1.55 (0.64–3.80)	0.34
Flat/unit/apartment in one-storey block	2.37 (1.89–2.98)	P < 0.001
Flat/unit/apartment in two-storey block	1.98 (1.59–2.48)	P < 0.001
Flat/unit/apartment in three-storey block	1.88 (1.45–2.44)	P < 0.001
Flat/unit/apartment in four to nine-storey block	1.38 (1.00–1.91)	0.05
Flat/unit/apartment in ten or more storey block	1.24 (0.74–2.07)	0.42
Flat/unit/apartment attached to a house	2.90 (1.93–4.35)	P < 0.001
Flat/unit/apartment attached to a shop, office etc	1.61 (0.71–3.66)	0.26
Caravan/Tent/Cabin/Houseboat	3.20 (1.81–5.64)	P < 0.001
*Body Mass Index*	1.00 (1.00–1.01)	0.86
*Aboriginal and Torres Strait Islander Status*		
Not of Indigenous Origin	1	
Aboriginal	1.14 (0.76–1.70)	0.53
Torres Strait Islander	1.57 (0.10–25.48)	0.75
Both Aboriginal and Torres Strait Islander	0.33 (0.05–2.04)	0.23

**Fig. 4:**
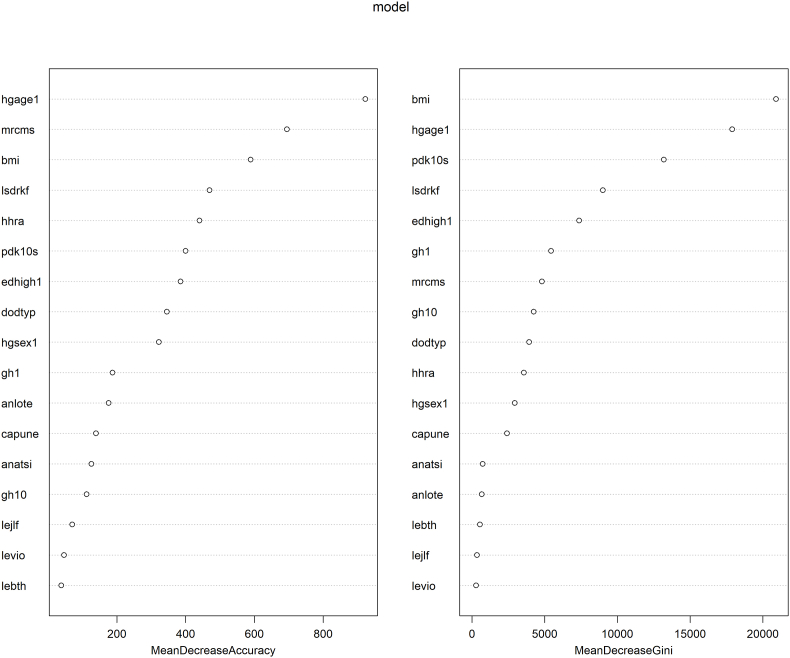
Importance of variables for random forest Model as per the Mean Decrease in Accuracy and Mean Decrease in Gini statistic upon the variable's exclusion from the model.* Caption: Variables with increasing importance are listed from top to bottom with their corresponding mean decrease in accuracy and mean decrease in Gini. Both are performance measures of importance. *Note: hgage1 is “Age at first follow-up”, mrcms is “Martial Status over time”, bmi “BMI over time”, lsdrkf “Alcohol Consumption over time”, hhra “Remoteness of Residence over time”, edhigh1 “Highest Educational Qualification over time”, pdk10s “K10 score over time”, dodtyp “Dwelling type over time”, hgsex1 “Gender at first followup”, gh1 “General Health and wellbeing over time (1–5)", anlote “Speaks Language other than English over time”, capune “Percent of time spent unemployed in the last financial year”, anatsi “Aboriginal and Torres Strait Islander status over time”, gh10 “Social functioning given health status (1–5) over time”, lejif “Member of family jailed in the last year”, levi “Victim of Physical Violence in the last year”, lebth “Birth/adoption of a new child".

More generally, the random forest model found that the seven most important variables that classified participants into one of the SEP or social mobility groups were BMI, age, marital status, psychological distress score, alcohol consumption, education level, and remoteness of residence gradient (Fig. 4). The balanced accuracy ranged from 89% for low SEP to 77% for decreasing SEP. The specificity (96%) and negative predictive values were very high (94%), but sensitivity (57%) and positive predictive values (69%) were modest. (Table 4) This implies that people not satisfying the criteria for decreasing SEP are unlikely to experience decreasing SEP, whereas there is a high level of uncertainty for people satisfying the criteria for decreasing SEP as to whether they actually experience decreasing SEP.

**Table 4:** Evaluation of random forest Model for Household Income Caption: Performance Measures for Random Forest Model in the classification of people by specific socio-economic position and rising and falling socio-economic position.

	High Income	Upper Middle Income	Lower Middle Income	Low Income	Rising Income (UM ->LM)	Falling Income (UM ->LM)
Sensitivity	74%	71%	64%	83%	62%	57%
Specificity	94%	88%	95%	95%	94%	96%
Pos Pred Value	71%	63%	71%	77%	66%	69%
Neg Pred Value	95%	91%	94%	97%	93%	94%
Prevalence	17%	22%	15%	16%	16%	13%
Detection Rate	13%	16%	9%	13%	10%	7%
Detection Prevalence	18%	25%	13%	17%	15%	11%
Balanced Accuracy	84%	80%	80%	89%	78%	77%

## 4 Discussion

This study investigated the association between SEP and social mobility (that is, change in SEP), and a range of physical health, mental health and treatment outcomes, in a population-based prospective cohort study in Australia. Overall, there was an increased risk of new onset chronic disease among lower SEP groups, evident for all reported conditions. Those experiencing rising or falling SEP had a risk of new onset chronic disease similar to their final SEP, rather than their initial SEP. That is, the risk of new onset chronic disease outcomes for the decreasing SEP group was similar to the lower middle-income SEP group, and not the upper middle SEP group. Similarly, those experiencing increasing SEP had a similar risk of new onset chronic disease outcomes to the upper middle SEP group, rather than the lower middle income. This pattern was evident for both physical health outcomes (with the exception of Type 1 diabetes and any cancer diagnosis) and mental health outcomes. No previous studies in the Australian context or globally, have shown the consistent effects of social mobility over wide range of physical and mental health outcomes over multiple decades in this detail.

Additional analyses suggested that older age, poorer social relationships, psychological distress, lower educational achievement and periods of unemployment, and type and area of residence were factors associated with a decreasing trajectory of SEP. The transition period of decreasing SEP was also found to be important in predicting the onset of the disease outcome, baseline ‘protective’ factors such as educational qualifications, diet and lifestyle, before the decline in SEP may not be preventive of subsequent illness. This suggests that it is current SEP which is associated with health outcomes rather than past SEP.

In contrast, there was limited evidence of a socio-economic gradient in self-reported initiation of treatment relating to both physical health and mental health outcomes. Overall, there was no substantial difference in treatment initiation between the lower SEP group, or between increasing or decreasing SEP groups, compared to the high SEP referent category, with the exception of treatments for arthritis or osteoporosis, and treatment for poor mental health. For arthritis or osteoporosis treatment commencement, those of lower SEP, and either decreasing or increasing SEP, were more likely to have commenced treatment earlier; whereas those with lower SEP and those of decreasing SEP, were more likely to visit a mental health clinician later. The less prominent social gradient for the initiation of treatment may reflect a health system that is nominally universal (under the Australian Medicare system), and which is broadly accessible across socio-economic position. However, this finding is not consistent with other Australian studies that show socio-economic differences in health service access by measures of other markers of socio-economic position such as socio-economic area of residence (Robards et al., 2019) and remoteness (Carey et al., 2013). The role of transitions in educational achievement, employment status and occupation and access to health care in the Australia context is less clear and is an area for future research.

There are several methodological considerations when interpreting the findings from the current study. Firstly, establishing the extent to which SEP is a putative ‘cause’ of a given health outcome is difficult to ascertain, and there are clearly bi-directional associations between SEP and health as has been previously noted (Hoven & Siegrist, 2013), and evidence of both social causation and social selection of health outcomes. However, a strength of the current study was the longitudinal study design, based on a representative sample of the Australian population. Analyses could clearly delineate temporal associations between a given SEP, or level of social mobility, and incident health outcomes allowing a determination of how SEP, or trajectory of SEP, was associated with the subsequent onset of selected disease outcomes. The HILDA cohort also contains a large set of variables to document the breadth and depth of social variables with some focus on health variables. This allowed for the incorporation of a range of confounders associated with social determinants of incident health outcomes.

Secondly, the definition of SEP and social mobility in the current study was limited to household income level. Income was selected as the main exposure measure given that it was asked of study participants at every follow-up for the last seventeen waves, allowing the construction of detailed trajectories over time. A limitation of k-longitudinal means is that only one continuous variable can be us used to identify trajectories, so annual household income was employed.

There is also potential measurement bias in both the main exposure and outcome variables, as data on SEP and health outcomes were based on self-report. For income level it may be participants under- or over-estimated their household income resulting in biased SEP-health outcome associations. Similarly, health outcomes were based on a self-reported diagnosis of selected outcomes (for example for diabetes, respiratory disease, and cardiovascular disease), and are potentially affected by recall bias. However, findings for mental health outcomes were consistent when comparing a self-reported diagnosis of depression or anxiety with a validated screening measure of psychological distress measure (the Kessler 10).

Findings are similar to previous research which has found improvements in improved cause-specific cancer mortality with improving SEP (Poulson et al., 2022), and studies showing the cumulative effects of low SEP on hypertension (Lopes et al., 2021). Similarly, previous studies have also shown healthy lifestyle factors (healthy selection bias) associated with rises in SEP, affecting subsequent health outcomes (Missinne et al., 2015), and rising SEP improved depressive symptoms and falling SEP increased depressive symptoms, mainly among men (Gugushvili et al., 2019). Less consistent findings were evident in specific sub-populations, for example for cardiovascular outcomes among African Americans where a rise in SEP increased risk of diabetic outcomes (Chen & Miller, 2022), among refugees who experienced more severe depressive symptoms post-migration with falling SEP (Euteneuer & Schafer, 2018), and increased anti-depressant use among those with falling SEP (Melchior et al., 2018).

Findings of the current study suggest that modifying SEP can have impacts on subsequent health outcomes and health inequalities. While disentangling the individual determinants of SEP is problematic, there are an array of potentially modifiable risk factors and sub-populations of relevance for specific policy responses. For example, education, unemployment, and housing, alcohol consumption, and specific sub-populations such as older age groups, those living alone, rural and remote populations, and Aboriginal and Torres Strait Islanders. Policy interventions that acknowledge the syndemics of health disparities and address the main determinants of social mobility could potentially prevent decreases in SEP, reducing socio-economic inequality and preventing poor outcomes across a range of physical and mental health conditions. Similarly, interventions to facilitate upward social mobility, such as early childhood education resources (Garon-Carrier et al., 2022), housing affordability (Wetzstein, 2017), education and training (Baum et al., 2011), structured employment support and income re-distribution (Knotz, 2018), and health service access (McMaughan et al., 2020), also have the potential to facilitate higher SEP and reduced socio-economic inequalities.

## 5 Conclusion

This study investigated the association between changes in socio-economic position (SEP) trajectories and the onset of physical health outcomes, mental health outcomes, and associated treatment commencement, and found strong associations between decreasing SEP and poor health outcomes and improving health outcomes with increasing SEP. This study identifies a range of associated socio-demographic and psychosocial determinants for these SEP trajectories. Preventing people from falling in SEP and assisting those with low SEP across the social determinants of health can reduce risk of new onset chronic disease, and these findings can be used to inform interventions and policy responses to prevent the emergence of health inequalities in the Australian context.

## Ethics approval

The authors assert that all procedures contributing to this work comply with the ethical standards of the relevant national and institutional committees. Approval to use the Household Income and Labour Dynamics (HILDA) data was obtained via the Department of Social Services Longitudinal Studies Dataverse [ https://dataverse.ada.edu.au/dataverse/DSSLongitudinalStudies] with approval number ref#756947.

## Author contributions

MD led the conception and design of the study, conducted the data analyses, and drafted the manuscript. AP contributed to the conception and design of the study, data interpretation, and critical revision of the manuscript. SS contributed to the study design, oversight of the statistical analysis, and provided critical revision of the manuscript. GU contributed to data interpretation and provided critical revision of the manuscript. CHS contributed to data interpretation, provided perspectives on policy implications of findings, and provided critical revisions of the manuscript. JE contributed to conception of the study and provision of critical revision of the manuscript.

## Funding

This work was supported by the Centre for Research Excellence for Integrated Health and Social Care (CREHSCI), funded by the 10.13039/501100000925National Health and Medical Research Council [APP1198477].

## Credit author statement

Mithilesh Dronavalli: Conceptualisation, Methodology, Software, Formal Analysis, Investigation, Data Curation, Writing - Original Drat, Writing - Review and Editing, Visualisation/Data Presentation, Project Administration.

Andrew Page: Conceptualisation, Methodology, Formal Analysis, Writing - Original Drat, Writing - Review and Editing, Supervision, Project Administration.

Sandro Sperandei: Conceptualisation, Methodology, Software, Validation, Formal Analysis, Writing - Original Drat, Writing - Review and Editing, Visualisation/Data Presentation, Supervision,

Gabriela Uribe: Conceptualisation, Writing - Original Drat, Writing - Review and Editing, Supervision,

Carmen Huckel Schneider: Conceptualisation, Writing - Original Drat, Writing - Review and Editing, Supervision,

John Eastwood: Conceptualisation, Writing - Original Drat, Writing - Review and Editing, Supervision.

## Declaration of competing interest

None declared.

## Data Availability

Data: https://dataverse.ada.edu.au/dataverse/DSSLongitudinalStudies | Code: All code Has been submitted as a supplement.
