# Supplementary material for: Determinants and health outcomes of trajectories of social mobility in Australia
Source: SSM Popul Health. 2023 Jan 5;21:101336. doi: 10.1016/j.ssmph.2023.101336 (PMC9843487; doi:10.1016/j.ssmph.2023.101336)
Supplement: Multimedia component 1 [file mmc1.pdf]

```
cd "C:\Users\mithi\OneDrive - Western Sydney University\HILDA20\MyCODE\6groups"

log using "C:\Users\mithi\OneDrive - Western Sydney University\HILDA20\Results\20_Datastep.smcl", replace


do "C:\Users\mithi\OneDrive - Western Sydney University\HILDA20\MyCODE\program13-longfile-release-20.do"

do "C:\Users\mithi\OneDrive - Western Sydney University\HILDA20\MyCODE\program16-widefile-release-20.do"

do "C:\Users\mithi\OneDrive - Western Sydney University\HILDA20\MyCODE\hiband extractor.do"


rscript using "C:\Users\mithi\OneDrive - Western Sydney University\HILDA20\MyCODE\6groups\6queue_7yrs.r"

rscript using "C:\Users\mithi\OneDrive - Western Sydney University\HILDA20\MyCODE\6groups\Hilda Graph clusters.r"


do "C:\Users\mithi\OneDrive - Western Sydney University\HILDA20\MyCODE\6groups\6_DataStep v6.do"
save "C:\Users\mithi\OneDrive - Western Sydney University\HILDA20\FinalData20\Cluster_Combi_HILDA.dta", replace

capture noisily log close

log using "C:\Users\mithi\OneDrive - Western Sydney University\HILDA20\Results\20_Analysis_step.smcl", replace

/*
gen num_clusters =1 if clusters_6 =="B"
recode num_clusters (.=2) if clusters_6 =="A"
recode num_clusters (.=3) if clusters_6 =="E"
recode num_clusters (.=4) if clusters_6 =="D"
recode num_clusters (.=5) if clusters_6 =="C"
recode num_clusters (.=6) if clusters_6 =="F"

*/


do "C:\Users\mithi\OneDrive - Western Sydney University\HILDA20\MyCODE\6groups\6_Mind v6.do"

do "C:\Users\mithi\OneDrive - Western Sydney University\HILDA20\MyCODE\6groups\6_Body v6.do"

do "C:\Users\mithi\OneDrive - Western Sydney University\HILDA20\MyCODE\6groups\6_FINAL MLOGIT Random Forest_ml_prep.do"

log close

rscript using "C:\Users\mithi\OneDrive - Western Sydney University\HILDA20\MyCODE\6groups\LOOPFP v10.r", force

rscript using "C:\Users\mithi\OneDrive - Western Sydney University\HILDA20\Results\UnifiedForestPlot\Serious Illness Final v1.r", force

rscript using "C:\Users\mithi\OneDrive - Western Sydney University\HILDA20\Results\UnifiedForestPlot\Treatment v1.r", force


rscript using "C:\Users\mithi\OneDrive - Western Sydney University\HILDA20\ml\FINAL\RandomForrest.r", force
```

```
*****************************************************************************
* Created by: Claire Sun                                                    *
* Updated by: Nicole Watson & Markus Hahn                                   *
*                                                                           *
* Purpose of the progam:                                                    *
* ======================                                                    *
* This program creates an unbalanced and a balanced longitudinal data file,    *
* using the the combined files. The new data files are in Stata's long format. *
* Please note that we use 'tempfile tempdata_w' to create a macro (local) that *
* allows us to access a temporary data file which will be automatically        *
* deleted when this do-file ends.                                           *
*****************************************************************************

clear
clear matrix
clear mata

set maxvar 120000

// Specify directories (use "." to point to current directory)
local origdatadir "C:\Users\mithi\OneDrive - Western Sydney University\HILDA20\UnFormattedData"  // Location of original HILDA data files
local newdatadir  "C:\Users\mithi\OneDrive - Western Sydney University\HILDA20\FinalData20"     // Location to which to write new data files


// SECTION 1: CREATING AN UNBALANCED DATASET (LONG-FORMAT)
// The following code uses the combined files. Since we want the final
// file to be in long format, we need to remove the alphabetic wave
// indicator from the variable names and a create a variable containing
// the numeric wave indicator. For that we use a loop to make things easier.
// Usually not all variables are required. Therefore, in the code we just
// select a few before saving the temporary data file.

//local varstokeep hwhmhl hhrhid hhrpid hhpxid hhresp hhstate hhsos ancob losathl ///
 //                wsce wscei wscef wscme wscmei wscmef wscoe wscoei wscoef

local i = 0
foreach w in a b c d e f g h i j k l m n o p q r s t {
    use "`origdatadir'\Combined_`w'200c"

    renpfix `w'      // Strip off wave prefix
    local i = `i'+1  // Increase (wave) counter by 1
    gen wave = `i'   // Create wave indicator (1, 2, ...)

    // select variables needed
    if ("`varstokeep'"!="") {
        local tokeep                                 // empty to keep list
        foreach var of v * {             // loop over all selected variables
            capture confirm variable `var'           // check whether variable exists in current wave
            if (!_rc) local tokeep `tokeep' `var'    // mark for inclusion if variable exists
            }
        keep xwaveid wave `tokeep' // keep selected variables
        }

    // Save temporary data file
    tempfile tempdata_`w'
    save "`tempdata_`w''"
}

// The following code appends the temporary data files for each wave to create
// an unbalanced panel.
clear
```

```
foreach w in a b c d e f g h i j k l m n o p q r s t {
    append using "`tempdata_`w''"
    }
order xwaveid wave
sort  xwaveid wave

// Save new data set
save "C:\Users\mithi\OneDrive - Western Sydney University\HILDA20\FinalData20\Combi_HILDA20.dta", replace


*BELOW NOT USED, no need for balanced data

/*
// SECTION 2: CREATING BALANCED DATASET (LONG-FORMAT)
// We prepare the master file because we want to keep some useful information.
// To create a balanced panel we can use the variable ivwptn which contains
// the interview pattern for each person. If you want keep more variables from
// the master file, you need to change to following code.

use "`origdatadir'\Master_t200c"
keep xwaveid ivwptn // You can keep more variables if you want
merge 1:m xwaveid using "`newdatadir'\long-file-unbalanced", nogen
keep if ivwptn=="XXXXXXXXXXXXXXXXXXXX" // only keep persons responding in every wave
order xwaveid wave
sort  xwaveid wave

// Save new data set
save "`newdatadir'\long-file-balanced", replace


*/
```

```
******************************************************************************
* Created by: Claire Sun                                                     *
* Updated by: Nicole Watson & Markus Hahn                                    *
*                                                                            *
* Purpose of the progam:                                                     *
* ======================                                                     *
* This program creates an unbalanced and a balanced longitudinal data file,  *
* using the the combined files. The new data files are in Stata's wide format. *
* Please note that we use 'tempfile tempdata_w' to create a macro (local) that *
* allows us to access a temporary data file which will be automatically      *
* deleted when this do-file ends.                                            *
******************************************************************************

clear
clear mata
clear matrix

set memory 10g
set maxvar 120000

// Specify directories (use "." to point to current directory)
local origdatadir "C:\Users\mithi\OneDrive - Western Sydney University\HILDA20\UnFormattedData" // Location of original HILDA data files
local newdatadir  "C:\Users\mithi\OneDrive - Western Sydney University\HILDA20\FinalData20"     // Location to which to write new data files

// SECTION 1: CREATING AN UNBALANCED DATASET (WIDE-FORMAT)
// The following code uses the combined files. We use a loop to make
// things easier.  Usually not all variables are required. Therefore, in
// the code we just select a few before saving the temporary data file.

// Specify variables you want to keep without wave prefix, i.e. hhrhid
// rather that ahhrhid.  If you want to include all variables, specify
// "local varstokeep", i.e. without variables.
//unab all_vars: *

//local varstokeep all_vars *

foreach w in a b c d e f g h i j k l m n o p q r s t {
    use "`origdatadir'\Combined_`w'200c.dta"

    // select variables needed
    if ("`varstokeep'"!="") {
        local tokeep                                 // empty to keep list
        foreach v  of var * {              // loop over all selected variables
            capture confirm variable `w'`var'        // check whether variable exists in current wave
            if (!_rc) local tokeep `tokeep' `w'`var' // mark for inclusion if variable exists
            }
        keep xwaveid `tokeep' // keep selected variables
        }

    // Save temporary data file
    tempfile tempdata_`w'
    save "`tempdata_`w''"
}

use "`origdatadir'\Master_t200c"
//keep xwaveid ivwptn // You can keep more variables if you want
sort xwaveid

foreach w in a b c d e f g h i j k l m n o p q r s t {
    merge 1:1 xwaveid using "`tempdata_`w''", nogen
}
```

```

save "`newdatadir'\wide-file-unbalanced", replace


// SECTION 2: CREATING BALANCED DATASET (WIDE-FORMAT)
// To create a balanced panel we can use the variable ivwptn which contains
// the interview pattern for each person.

use "`newdatadir'\wide-file-unbalanced"
keep if ivwptn=="XXXXXXXXXXXXXXXXXXXX"
save "`newdatadir'\wide-file-balanced", replace


```

```
clear
clear mata
clear matrix

set memory 10g
set maxvar 120000

use "C:\Users\mithi\OneDrive - Western Sydney University\HILDA20\FinalData20\wide-file-unbalanced.dta"

mdesc  dhiband ehiband fhiband2 ghiband2 hhiband2 ihiband3 jhiband3 khiband3 lhiband3 mhiband3 nhiband3 ohiband3 phiband3 qhiband3 rhiband3 shiband3 thiband3
gen real_xwaveid = real(xwaveid)
keep real_xwaveid  dhiband ehiband fhiband2 ghiband2 hhiband2 ihiband3 jhiband3 khiband3 lhiband3 mhiband3 nhiband3 ohiband3 phiband3 qhiband3 rhiband3 shiband3 thiband3
rename dhiband dhiband3
rename ehiband ehiband3
rename fhiband fhiband3
rename ghiband ghiband3
rename hhiband hhiband3


rename dhiband3 hiband31
rename ehiband3 hiband32
rename fhiband3 hiband33
rename ghiband3 hiband34
rename hhiband3 hiband35
rename ihiband3 hiband36
rename jhiband3 hiband37
rename khiband3 hiband38
rename lhiband3 hiband39
rename mhiband3 hiband310
rename nhiband3 hiband311
rename ohiband3 hiband312
rename phiband3 hiband313
rename qhiband3 hiband314
rename rhiband3 hiband315
rename shiband3 hiband316
rename thiband3 hiband317

reshape long hiband3, i(real_xwaveid) j(wave)

replace hiband* = . if hiband <=0




xtset real wave
bysort real_xwaveid: ipolate hiband3 wave, gen(ipol_hiband3)
codebook ipol_hiband3 hiband3
generate round_ipol = round(ipol,0)
codebook round_ipol
list round_ipol
drop ipol_hiband3 hiband3
rename round_ipol hiband3
reshape wide

save "C:\Users\mithi\OneDrive - Western Sydney University\HILDA20\FinalData20\min_Combi_Hilda.dta", replace
```

```
#install.packages("kml")
#install.packages("kml3d")
#install.packages("ParLongData")

#install.packages("tiff")



library(kml)
library(kml3d)
library(haven)
#library(ParLongData)
library(tiff)




q=6




set.seed(1)

hiband3 <- read_dta("C:/Users/mithi/OneDrive - Western Sydney University/
HILDA20/FinalData20/min_Combi_Hilda.dta")


hiband3_m<-as.matrix(hiband3[, 2:18])  #COnverting the data to a matrix

cldSDQ <- cld(hiband3_m, timeInData=1:17, maxNA = 10) #Converting the matrix
into a data_blob for kml to use

kml(cldSDQ,nbClusters = q,nbRedrawing = 25)
#X11(type="Xlib")

hiband3$clusters <- getClusters(cldSDQ, q)

#choice(cldSDQ, savePlot(filename = "6clusters", type = "tiff"))
#write.csv(hiband3, "C:/Users/mithi/OneDrive - Western Sydney University/
HILDA20/FinalData20/HILDAhiband3_6.csv")

#part <- partition(rep(c(1,2,3,4,5,6),time=10))

  #par("tck"=2)

tiff("C:/Users/mithi/OneDrive - Western Sydney University/HILDA20/Results/
6clusters_HILDA20.tiff", units="in", width=10, height=8, res=300)
  plot(cldSDQ,q,parMean=parMEAN(type="l"),addLegend = TRUE ,
,parTraj=parTRAJ(type="n") , xaxt = "n",  yaxt = "n", toPlot="traj",ylab =
"Household Income ($,000)", xlab = "Years in the HILDA Cohort")
```

```
  # X-axis
  axis(1,
     tck=1,
       col = "gray", lty=2, at =
c(0,1,2,3,4,5,6,7,8,9,10,11,12,13,14,15,16,17))

  # Y-axis
  y = c("No
Income","10","20","30","40","50","60","80","100","125","150","200","")
  axis(2,
     tck=1,
       col = "gray", lty=2, at = seq_along(y),labels = y,par(las=0))

  #title(main="Social Mobility Clusters over 10 years from the HILDA 20 Cohort
", sub="Clusters generated by Longitudinal K-Means")




  #grid(nx = 4, # X-axis divided in two sections
   #  ny = 6, # Y-axis divided in three sections
    # lty = 2, col = "gray", lwd = 1)


  dev.off()

  pdf("C:/Users/mithi/OneDrive - Western Sydney University/HILDA20/Results/
6clusters_HILDA20.pdf",  width=13.33, height=7.5)
  plot(cldSDQ,q,parMean=parMEAN(type="l"),addLegend = TRUE ,
,parTraj=parTRAJ(type="n") , xaxt = "n",  yaxt = "n", toPlot="traj",ylab =
"Household Income ($,000)", xlab = "Years in the HILDA Cohort")


  # X-axis
  axis(1,
       tck=1,
       col = "gray", lty=2, at =
c(0,1,2,3,4,5,6,7,8,9,10,11,12,13,14,15,16,17))

  # Y-axis
  y = c("No
Income","10","20","30","40","50","60","80","100","125","150","200","")
  axis(2,
       tck=1,
       col = "gray", lty=2, at = seq_along(y),labels = y,par(las=0))

  #title(main="Social Mobility Clusters over 10 years from the HILDA 20 Cohort
", sub="Clusters generated by Longitudinal K-Means")
```

```
#grid(nx = 4, # X-axis divided in two sections
#  ny = 6, # Y-axis divided in three sections
# lty = 2, col = "gray", lwd = 1)


dev.off()
```

```
rm(list=ls())

library(tidyverse)
library(lubridate)




load("C:/Users/mithi/OneDrive - Western Sydney University/HILDA20/FinalData20/
HILDAcdlSQ6.RData")

Mith = hiband3 %>%
  filter(!is.na(clusters_6)) %>%
  group_by(clusters_6) %>%
  summarise(AV_1 = mean(hiband31, na.rm=T),
            AV_2 = mean(hiband32, na.rm=T),
            AV_3 = mean(hiband33, na.rm=T),
            AV_4 = mean(hiband34, na.rm=T),
            AV_5 = mean(hiband35, na.rm=T),
            AV_6 = mean(hiband36, na.rm=T),
            AV_7 = mean(hiband37, na.rm=T),
            AV_8 = mean(hiband38, na.rm=T),
            AV_9 = mean(hiband39, na.rm=T),
            AV_10 = mean(hiband310, na.rm=T),
            AV_11 = mean(hiband311, na.rm=T),
            AV_12 = mean(hiband312, na.rm=T),
            AV_13 = mean(hiband313, na.rm=T),
            AV_14 = mean(hiband314, na.rm=T),
            AV_15 = mean(hiband315, na.rm=T),
            AV_16 = mean(hiband316, na.rm=T),
            AV_17 = mean(hiband317, na.rm=T),

            LCI_1 = AV_1 + qt(0.025, n()-1) * sd(hiband31, na.rm=T) /
sqrt(n()),
            LCI_2 = AV_2 + qt(0.025, n()-1) * sd(hiband32, na.rm=T) /
sqrt(n()),
            LCI_3 = AV_3 + qt(0.025, n()-1) * sd(hiband33, na.rm=T) /
sqrt(n()),
            LCI_4 = AV_4 + qt(0.025, n()-1) * sd(hiband34, na.rm=T) /
sqrt(n()),
            LCI_5 = AV_5 + qt(0.025, n()-1) * sd(hiband35, na.rm=T) /
sqrt(n()),
            LCI_6 = AV_6 + qt(0.025, n()-1) * sd(hiband36, na.rm=T) /
sqrt(n()),
            LCI_7 = AV_7 + qt(0.025, n()-1) * sd(hiband37, na.rm=T) /
sqrt(n()),
            LCI_8 = AV_8 + qt(0.025, n()-1) * sd(hiband38, na.rm=T) /
sqrt(n()),
            LCI_9 = AV_9 + qt(0.025, n()-1) * sd(hiband39, na.rm=T) /
sqrt(n()),
            LCI_10 = AV_10 + qt(0.025, n()-1) * sd(hiband310, na.rm=T) /
sqrt(n()),
            LCI_11 = AV_11 + qt(0.025, n()-1) * sd(hiband311, na.rm=T) /
sqrt(n()),
```

```
            LCI_12 = AV_12 + qt(0.025, n()-1) * sd(hiband312, na.rm=T) /
sqrt(n()),
            LCI_13 = AV_13 + qt(0.025, n()-1) * sd(hiband313, na.rm=T) /
sqrt(n()),
            LCI_14 = AV_14 + qt(0.025, n()-1) * sd(hiband314, na.rm=T) /
sqrt(n()),
            LCI_15 = AV_15 + qt(0.025, n()-1) * sd(hiband315, na.rm=T) /
sqrt(n()),
            LCI_16 = AV_16 + qt(0.025, n()-1) * sd(hiband316, na.rm=T) /
sqrt(n()),
            LCI_17 = AV_17 + qt(0.025, n()-1) * sd(hiband317, na.rm=T) /
sqrt(n()),

            UCI_1 = AV_1 + qt(0.975, n()-1) * sd(hiband31, na.rm=T) /
sqrt(n()),
            UCI_2 = AV_2 + qt(0.975, n()-1) * sd(hiband32, na.rm=T) /
sqrt(n()),
            UCI_3 = AV_3 + qt(0.975, n()-1) * sd(hiband33, na.rm=T) /
sqrt(n()),
            UCI_4 = AV_4 + qt(0.975, n()-1) * sd(hiband34, na.rm=T) /
sqrt(n()),
            UCI_5 = AV_5 + qt(0.975, n()-1) * sd(hiband35, na.rm=T) /
sqrt(n()),
            UCI_6 = AV_6 + qt(0.975, n()-1) * sd(hiband36, na.rm=T) /
sqrt(n()),
            UCI_7 = AV_7 + qt(0.975, n()-1) * sd(hiband37, na.rm=T) /
sqrt(n()),
            UCI_8 = AV_8 + qt(0.975, n()-1) * sd(hiband38, na.rm=T) /
sqrt(n()),
            UCI_9 = AV_9 + qt(0.975, n()-1) * sd(hiband39, na.rm=T) /
sqrt(n()),
            UCI_10 = AV_10 + qt(0.975, n()-1) * sd(hiband310, na.rm=T) /
sqrt(n()),
            UCI_11 = AV_11 + qt(0.975, n()-1) * sd(hiband311, na.rm=T) /
sqrt(n()),
            UCI_12 = AV_12 + qt(0.975, n()-1) * sd(hiband312, na.rm=T) /
sqrt(n()),
            UCI_13 = AV_13 + qt(0.975, n()-1) * sd(hiband313, na.rm=T) /
sqrt(n()),
            UCI_14 = AV_14 + qt(0.975, n()-1) * sd(hiband314, na.rm=T) /
sqrt(n()),
            UCI_15 = AV_15 + qt(0.975, n()-1) * sd(hiband315, na.rm=T) /
sqrt(n()),
            UCI_16 = AV_16 + qt(0.975, n()-1) * sd(hiband316, na.rm=T) /
sqrt(n()),
            UCI_17 = AV_17 + qt(0.975, n()-1) * sd(hiband317, na.rm=T) /
sqrt(n())) %>%
  pivot_longer(-clusters_6,
               names_to = c("Measure","Time"),
               names_pattern = "(.*)_(.*)",
               values_to = "Value") %>%
  pivot_wider(c(clusters_6,Time),
              names_from = Measure,
              values_from = Value) %>%
```

```
  mutate(Time = as.numeric(Time),
         clusters_6 = factor(clusters_6,
                             levels = c("B","A","E",
                               "C","D","F")
                             ,

                             labels = c("High (17.7%)","Upper-Middle (21.6%)",
                                        "Lower-Middle: (15.1%)","Low (16.3%)",
                                        "Increasing (17.2%)",
                                        "Decreasing (11.9%)"))) %>%
  data.frame()

Num_cluster<- ggplot(Mith, aes(x = Time, y = AV, col = clusters_6)) +
  geom_smooth(method = 'loess', lwd = 1.25) +
   theme_bw() +
  labs(x = "Years in the HILDA cohort",
       y = "Household income ($,000)",
       col = "Trajectories, n = 16,187,(%n)",
       title = "Trajectories of House-hold income in the HILDA Cohort",
       caption = "Trajectories Derived using K-Longitudinal Means. Sample Size
calculated at year 6 (Wave 9)") +
  scale_y_continuous( limits=c(3,13), breaks = c(4, 5, 6, 7, 8,  9, 10, 11,
12),
                     labels = c(30,40,50,60,80,100,125,150,200))+
scale_x_continuous( limits=c(1,17), breaks = c(3,6, 9,12,15,18),
                    labels = c(3,6, 9,12,15,18))

[10] $100,000 - $124,999 668
[11] $125,000 - $149,999 495
[12] $150,000 - $199,999 447
[13] $200,000 or more 312
[1] Negative or Zero income 18
[2] $1 - $9,999 106
[3] $10,000 - $19,999 797
[4] $20,000 - $29,999 841
[5] $30,000 - $39,999 623
[6] $40,000 - $49,999 605
[7] $50,000 - $59,999 533
[8] $60,000 - $79,999 774
[9] $80,000 - $99,999

TiffFile="C:/Users/mithi/OneDrive - Western Sydney University/HILDA20/Results/
Num_Clusters.tiff"
tiff(TiffFile, units="in", width=14, height=9, res=300)
print(Num_cluster)
dev.off()
```

```
clear
clear matrix
clear mata
set maxvar 10000

set rmsg on
import delimited "C:\Users\mithi\OneDrive - Western Sydney University\HILDA20\FinalData20\HILDAhiband3_6.csv"
save "C:\Users\mithi\OneDrive - Western Sydney University\HILDA20\FinalData20\hiband3clusters_xwaveidnum.dta", replace
use "C:\Users\mithi\OneDrive - Western Sydney University\HILDA20\FinalData20\Combi_HILDA20.dta"


*drop xwaveid2
*gen xwaveid2 = xwaveid
*drop xwaveid2
 capture noisily gen real_xwaveid = real(xwaveid)
save "C:\Users\mithi\OneDrive - Western Sydney University\HILDA20\FinalData20\Combi_HILDA20.dta", replace
use "C:\Users\mithi\OneDrive - Western Sydney University\HILDA20\FinalData20\hiband3clusters_xwaveidnum.dta", clear

merge 1:m real_xwaveid using "C:\Users\mithi\OneDrive - Western Sydney University\HILDA20\FinalData20\Combi_HILDA20.dta"
//drop num_clusters
gen num_clusters =1 if clusters_6 =="B"
recode num_clusters (.=2) if clusters_6 =="A"
recode num_clusters (.=3) if clusters_6 =="E"
recode num_clusters (.=4) if clusters_6 =="D"
recode num_clusters (.=5) if clusters_6 =="C"
recode num_clusters (.=6) if clusters_6 =="F"




gen age_init = hgage1
by real_xwave (age_init), sort: replace age_init = age_init[1]

gen age_cat = 1 if age_init >0 & age_init <=18
recode age_cat (.=2) if age_init>18 & age_init <=30
recode age_cat (.=3) if age_init>30 & age_init <=45
recode age_cat (.=4) if age_init>45 & age_init <=65
recode age_cat (.=5) if age_init>65

xtset real_xwave wave

gen u18 = 1 if age_cat == 1
recode u18 (.=0) if age_cat !=.

//replace hepmanx = . if hepmanx <0

//replace hepmdep = . if hepmdep <0

//replace heanx = . if heanx <0
gen real_hhrhid = real(hhrhid)


save "C:\Users\mithi\OneDrive - Western Sydney University\HILDA20\FinalData20\Cluster_Combi_HILDA.dta", replace


keep  pdk10s bmi  num_clusters hgage1 hgsex1 hhra edhigh1 mrcms   anbcob  capune wave real_xwave heany heart hepmart hecan hepmcan hecbe hepmcbe hepmast hedi1 hepmdi1 hedi2 hepmdi2 hehbp hepmhbp
```

```
hehcd hepmhd heoc hepmoc hedep hepmdep heomi  hepmomi hecpmhp anlote hhrhid lsdrkf num_clusters
hgage1 hgsex1 gh1 gh10 edhigh1 mrcms capune anlote lsdrkf hhra   lejlf levio  lebth dodtyp bmi
anatsi heast

local y pdk10s bmi  num_clusters hgage1 hgsex1 hhra edhigh1 mrcms   anbcob  capune wave real_xwave
heany heart hepmart hecan hepmcan hecbe hepmcbe hepmast hedi1 hepmdi1 hedi2 hepmdi2 hehbp hepmhbp
hehcd hepmhd heoc hepmoc  hedep hepmdep heomi  hepmomi hecpmhp anlote hhrhid lsdrkf num_clusters
hgage1 hgsex1 gh1 gh10 edhigh1 mrcms capune anlote lsdrkf hhra   lejlf levio  lebth dodtyp bmi
anatsi heast

foreach y in `y'{
 capture noisily   replace `y' = . if `y' < 0
}


save "C:\Users\mithi\OneDrive - Western Sydney University\HILDA20\FinalData20\ConciseHILDA.dta",
replace


use "C:\Users\mithi\OneDrive - Western Sydney University\HILDA20\FinalData20\ConciseHILDA.dta",
replace
xtset real_xwave wave
 by real_xwave (wave): carryforward heany , replace
 by real_xwave (wave): carryforward heart , replace

 by real_xwave (wave): carryforward hecan , replace
 by real_xwave (wave): carryforward hecbe , replace
 by real_xwave (wave): carryforward hedep , replace
 by real_xwave (wave): carryforward hedi1 , replace
 by real_xwave (wave): carryforward hedi2 , replace
 by real_xwave (wave): carryforward hedi2 , replace
 by real_xwave (wave): carryforward hehbp , replace
 by real_xwave (wave): carryforward hehcd, replace

 by real_xwave (wave): carryforward heoc, replace
 by real_xwave (wave): carryforward heomi , replace
 //by real_xwave (wave): carryforward hepmanx , replace
 by real_xwave (wave): carryforward hepmart , replace
 by real_xwave (wave): carryforward hepmast , replace
 by real_xwave (wave): carryforward hepmcbe , replace
 by real_xwave (wave): carryforward hepmdep , replace
 by real_xwave (wave): carryforward hepmdi1 , replace
 by real_xwave (wave): carryforward hepmdi2 , replace



 by real_xwave (wave): carryforward bmi, replace
 by real_xwave (wave): carryforward edhigh1 , replace
 by real_xwave (wave): carryforward mrcms , replace
 by real_xwave (wave): carryforward anbcob , replace
 by real_xwave (wave): carryforward capune , replace
 by real_xwave (wave): carryforward pdk10s, replace
 by real_xwave (wave): carryforward anlote , replace
 by real_xwave (wave): carryforward lsdrkf , replace
 by real_xwave (wave): carryforward gh1 , replace
 by real_xwave (wave): carryforward gh10, replace

 by real_xwave (wave): carryforward lejlf, replace
 by real_xwave (wave): carryforward levio , replace
 by real_xwave (wave): carryforward lebth , replace
 by real_xwave (wave): carryforward dodtyp , replace
 by real_xwave (wave): carryforward anatsi , replace
```

```
 gen negwave = -wave



 bysort real_xwave (negwave): carryforward anatsi , back replace

 bysort real_xwave (negwave): carryforward anlote , back replace

 bysort real_xwave (negwave): carryforward anatsi , back replace


gen htn_hd_pvd_stroke = 1 if (heoc ==1 | hehcd ==1 | hehbp ==1) & wave==9
recode htn_hd_pvd_stroke (.=0) if (heoc ==0 | hehcd ==0 | hehbp ==0) & wave==9
recode htn_hd_pvd_stroke (. = 1) if (heoc ==1 | hehcd ==1 | hehbp ==1) & wave==10
recode htn_hd_pvd_stroke (. = 1) if (heoc ==1 | hehcd ==1 | hehbp ==1) & wave==11
recode htn_hd_pvd_stroke (. = 1) if (heoc ==1 | hehcd ==1 | hehbp ==1) & wave==12
recode htn_hd_pvd_stroke (. = 1) if (heoc ==1 | hehcd ==1 | hehbp ==1) & wave==13
recode htn_hd_pvd_stroke (. = 1) if (heoc ==1 | hehcd ==1 | hehbp ==1) & wave==14
recode htn_hd_pvd_stroke (. = 1) if (heoc ==1 | hehcd ==1 | hehbp ==1) & wave==15
recode htn_hd_pvd_stroke (. = 1) if (heoc ==1 | hehcd ==1 | hehbp ==1) & wave==16
recode htn_hd_pvd_stroke (. = 1) if (heoc ==1 | hehcd ==1 | hehbp ==1) & wave==17
recode htn_hd_pvd_stroke (. = 0) if (heoc ==0 | hehcd ==0 | hehbp ==0) & wave==10
recode htn_hd_pvd_stroke (. = 0) if (heoc ==0 | hehcd ==0 | hehbp ==0) & wave==11
recode htn_hd_pvd_stroke (. = 0) if (heoc ==0 | hehcd ==0 | hehbp ==0) & wave==12
recode htn_hd_pvd_stroke (. = 0) if (heoc ==0 | hehcd ==0 | hehbp ==0) & wave==13
recode htn_hd_pvd_stroke (. = 0) if (heoc ==0 | hehcd ==0 | hehbp ==0) & wave==14
recode htn_hd_pvd_stroke (. = 0) if (heoc ==0 | hehcd ==0 | hehbp ==0) & wave==15
recode htn_hd_pvd_stroke (. = 0) if (heoc ==0 | hehcd ==0 | hehbp ==0) & wave==16
recode htn_hd_pvd_stroke (. = 0) if (heoc ==0 | hehcd ==0 | hehbp ==0) & wave==17

gen treat_htn_hd_pvd_stroke = 1 if (hepmoc ==1 | hepmhd ==1 | hepmhbp ==1) & wave==9
recode treat_htn_hd_pvd_stroke (.=0) if (hepmoc ==0 | hepmhd ==0 | hepmhbp ==0) & wave==9
recode treat_htn_hd_pvd_stroke (. = 1) if (hepmoc ==1 | hepmhd ==1 | hepmhbp ==1) & wave==10
recode treat_htn_hd_pvd_stroke (. = 1) if (hepmoc ==1 | hepmhd ==1 | hepmhbp ==1) & wave==11
recode treat_htn_hd_pvd_stroke (. = 1) if (hepmoc ==1 | hepmhd ==1 | hepmhbp ==1) & wave==12
recode treat_htn_hd_pvd_stroke (. = 1) if (hepmoc ==1 | hepmhd ==1 | hepmhbp ==1) & wave==13
recode treat_htn_hd_pvd_stroke (. = 1) if (hepmoc ==1 | hepmhd ==1 | hepmhbp ==1) & wave==14
recode treat_htn_hd_pvd_stroke (. = 1) if (hepmoc ==1 | hepmhd ==1 | hepmhbp ==1) & wave==15
recode treat_htn_hd_pvd_stroke (. = 1) if (hepmoc ==1 | hepmhd ==1 | hepmhbp ==1) & wave==16
recode treat_htn_hd_pvd_stroke (. = 1) if (hepmoc ==1 | hepmhd ==1 | hepmhbp ==1) & wave==17
recode treat_htn_hd_pvd_stroke (. = 0) if (hepmoc ==0 | hepmhd ==0 | hepmhbp ==0) & wave==10
recode treat_htn_hd_pvd_stroke (. = 0) if (hepmoc ==0 | hepmhd ==0 | hepmhbp ==0) & wave==11
recode treat_htn_hd_pvd_stroke (. = 0) if (hepmoc ==0 | hepmhd ==0 | hepmhbp ==0) & wave==12
recode treat_htn_hd_pvd_stroke (. = 0) if (hepmoc ==0 | hepmhd ==0 | hepmhbp ==0) & wave==13
recode treat_htn_hd_pvd_stroke (. = 0) if (hepmoc ==0 | hepmhd ==0 | hepmhbp ==0) & wave==14
recode treat_htn_hd_pvd_stroke (. = 0) if (hepmoc ==0 | hepmhd ==0 | hepmhbp ==0) & wave==15
recode treat_htn_hd_pvd_stroke (. = 0) if (hepmoc ==0 | hepmhd ==0 | hepmhbp ==0) & wave==16
```

```
recode treat_htn_hd_pvd_stroke (. = 0) if (hepmoc ==0 | hepmhd ==0 | hepmhbp ==0) & wave==17


gen any_mental_illness = 1 if (hedep ==1 | heomi ==1 ) & wave==9
recode any_mental_illness (.=0) if (hedep ==0 | heomi ==0 ) & wave==9
recode any_mental_illness (. = 1) if (hedep ==1 | heomi ==1 ) & wave==10
recode any_mental_illness (. = 1) if (hedep ==1 | heomi ==1 ) & wave==11
recode any_mental_illness (. = 1) if (hedep ==1 | heomi ==1 ) & wave==12
recode any_mental_illness (. = 1) if (hedep ==1 | heomi ==1 ) & wave==13
recode any_mental_illness (. = 1) if (hedep ==1 | heomi ==1 ) & wave==14
recode any_mental_illness (. = 1) if (hedep ==1 | heomi ==1 ) & wave==15
recode any_mental_illness (. = 1) if (hedep ==1 | heomi ==1 ) & wave==16
recode any_mental_illness (. = 1) if (hedep ==1 | heomi ==1 ) & wave==17
recode any_mental_illness (. = 0) if (hedep ==0 | heomi ==0 ) & wave==10
recode any_mental_illness (. = 0) if (hedep ==0 | heomi ==0 ) & wave==11
recode any_mental_illness (. = 0) if (hedep ==0 | heomi ==0 ) & wave==12
recode any_mental_illness (. = 0) if (hedep ==0 | heomi ==0 ) & wave==13
recode any_mental_illness (. = 0) if (hedep ==0 | heomi ==0 ) & wave==14
recode any_mental_illness (. = 0) if (hedep ==0 | heomi ==0 ) & wave==15
recode any_mental_illness (. = 0) if (hedep ==0 | heomi ==0 ) & wave==16
recode any_mental_illness (. = 0) if (hedep ==0 | heomi ==0 ) & wave==17

gen treat_any_mental_illness = 1 if (hepmdep ==1 | hepmomi ==1 | hecpmhp ==1) & wave==9
recode treat_any_mental_illness (.=0) if (hepmdep ==0 | hepmomi ==0 | hecpmhp ==0) & wave==9
recode treat_any_mental_illness (. = 1) if (hepmdep ==1 | hepmomi ==1 | hecpmhp ==1) & wave==10
recode treat_any_mental_illness (. = 1) if (hepmdep ==1 | hepmomi ==1 | hecpmhp ==1) & wave==11
recode treat_any_mental_illness (. = 1) if (hepmdep ==1 | hepmomi ==1 | hecpmhp ==1) & wave==12
recode treat_any_mental_illness (. = 1) if (hepmdep ==1 | hepmomi ==1 | hecpmhp ==1) & wave==13
recode treat_any_mental_illness (. = 1) if (hepmdep ==1 | hepmomi ==1 | hecpmhp ==1) & wave==14
recode treat_any_mental_illness (. = 1) if (hepmdep ==1 | hepmomi ==1 | hecpmhp ==1) & wave==15
recode treat_any_mental_illness (. = 1) if (hepmdep ==1 | hepmomi ==1 | hecpmhp ==1) & wave==16
recode treat_any_mental_illness (. = 1) if (hepmdep ==1 | hepmomi ==1 | hecpmhp ==1) & wave==17
recode treat_any_mental_illness (. = 0) if (hepmdep ==0 | hepmomi ==0 | hecpmhp ==0) & wave==10
recode treat_any_mental_illness (. = 0) if (hepmdep ==0 | hepmomi ==0 | hecpmhp ==0) & wave==11
recode treat_any_mental_illness (. = 0) if (hepmdep ==0 | hepmomi ==0 | hecpmhp ==0) & wave==12
recode treat_any_mental_illness (. = 0) if (hepmdep ==0 | hepmomi ==0 | hecpmhp ==0) & wave==13
recode treat_any_mental_illness (. = 0) if (hepmdep ==0 | hepmomi ==0 | hecpmhp ==0) & wave==14
recode treat_any_mental_illness (. = 0) if (hepmdep ==0 | hepmomi ==0 | hecpmhp ==0) & wave==15
recode treat_any_mental_illness (. = 0) if (hepmdep ==0 | hepmomi ==0 | hecpmhp ==0) & wave==16
recode treat_any_mental_illness (. = 0) if (hepmdep ==0 | hepmomi ==0 | hecpmhp ==0) & wave==17

gen actual_wave = wave -3
replace actual_wave =. if actual <=0
egen max_fup = max(actual_wave), by(real_xwaveid)
egen min_wave = min(wave), by(real_xwaveid)
gen wave9 = 1 if wave ==9
xtset real_xwave wave
 by real_xwave (wave): carryforward wave9 , replace
 bysort real_xwave (negwave): carryforward wave9 , back replace
save "C:\Users\mithi\OneDrive - Western Sydney University\HILDA20\FinalData20\ConciseHILDA.dta",
replace

```

```
clear
clear mata
clear matrix

set maxvar 120000

use "C:\Users\mithi\OneDrive - Western Sydney University\HILDA20\FinalData20\ConciseHILDA.dta", replace


//menbreg  pdk10s i.num_clusters  c.wave c.age_init, exposure(wave) || hhrhid: , irr nolog //no significant interaction

menbreg  pdk10s i.num_clusters c.hgage1 i.hgsex1  i.anlote  c.capune i.hhra  i.mrcms i.anatsi i.hhra c.edhigh1 i.lsdrkf , exposure(wave) || hhrhid: , irr nolog
putexcel set "C:\Users\mithi\OneDrive - Western Sydney University\HILDA20\MyCODE\Forest Plots\Changes in K10(Distress) over time.xlsx", replace

putexcel A1=matrix(r(table))

/*
poisson heanx i.num_cluster c.age_init, nolog irr
putexcel set "C:\Users\mithi\OneDrive - Western Sydney University\HILDA20\MyCODE\Forest Plots\heanx.xlsx", replace
putexcel A1=matrix(r(table))

poisson hedpn i.num_cluster  c.age_init, nolog irr
putexcel set "C:\Users\mithi\OneDrive - Western Sydney University\HILDA20\MyCODE\Forest Plots\hedpn.xlsx", replace
putexcel A1=matrix(r(table))


poisson hepmanx i.num_cluster  c.age_init, nolog irr
 putexcel set "C:\Users\mithi\OneDrive - Western Sydney University\HILDA20\MyCODE\Forest Plots\hepmanx.xlsx", replace
putexcel A1=matrix(r(table))

poisson hepmdpn i.num_cluster  c.age_init, nolog irr
putexcel set "C:\Users\mithi\OneDrive - Western Sydney University\HILDA20\MyCODE\Forest Plots\hepmdpn.xlsx", replace
putexcel A1=matrix(r(table))
*/










*Diagnosed with serious illness - Depression or anxiety

stset wave, id(real_xwave) failure(hedep==1)
 stcox i.num_cluster  c.hgage1 i.hgsex1  i.anlote  , tvc(c.capune i.hhra  i.mrcms i.anatsi i.hhra c.edhigh1 i.lsdrkf   )
 stcoxkm, by(num_cluster) separate
 *stphplot, by(num_cluster)  adjust(c.hgage1 i.hgsex1  i.anlote  c.capune i.hhra  i.mrcms i.anatsi i.hhra c.edhigh1 i.lsdrkf)
putexcel set "C:\Users\mithi\OneDrive - Western Sydney University\HILDA20\MyCODE\Forest Plots\hedep.xlsx", replace
putexcel A1=matrix(r(table))

```

```
*Takes prescription medication for - Depression or anxiety

xtpoisson hepmdep i.num_cluster  c.hgage1 i.hgsex1  i.anlote  c.capune i.hhra  i.mrcms i.hhra c.edhigh1 i.lsdrkf    if hedep ==1, irr


putexcel set "C:\Users\mithi\OneDrive - Western Sydney University\HILDA20\MyCODE\Forest Plots\hepmdep.xlsx", replace
putexcel A1=matrix(r(table))

*   Diagnosed with serious illness - Other mental illness

stset wave, id(real_xwave) failure(heomi==1)
stcox i.num_cluster  c.hgage1 i.hgsex1  i.anlote  , tvc(c.capune i.hhra  i.mrcms i.anatsi i.hhra c.edhigh1 i.lsdrkf   )
stcoxkm, by(num_cluster) separate
*stphplot, by(num_cluster)  adjust(c.hgage1 i.hgsex1  i.anlote  c.capune i.hhra  i.mrcms i.anatsi i.hhra c.edhigh1 i.lsdrkf)

putexcel set "C:\Users\mithi\OneDrive - Western Sydney University\HILDA20\MyCODE\Forest Plots\heomi.xlsx", replace
putexcel A1=matrix(r(table))

*Takes prescription medication for - Other mental illness

*xtpoisson hepmomi i.num_cluster  c.hgage1 i.hgsex1  i.anlote  c.capune i.hhra  i.mrcms i.hhra c.edhigh1 i.lsdrkf    if heomi ==1, irr


*putexcel set "C:\Users\mithi\OneDrive - Western Sydney University\HILDA20\MyCODE\Forest Plots\hepmomi.xlsx", replace
*putexcel A1=matrix(r(table))






////////////////////////////////////////////////////////////////////////////////

*   First seen psychologist /psychiatrist

stset wave if hedep==1 | heomi ==1, id(real_xwave) failure(hecpmhp==1)
stcox i.num_cluster  c.hgage1 i.hgsex1  i.anlote if hedep ==1 | heomi==1 , tvc(c.capune i.hhra  i.mrcms i.anatsi i.hhra c.edhigh1 i.lsdrkf   )
stcoxkm, by(num_cluster) separate
*stphplot, by(num_cluster)  adjust(c.hgage1 i.hgsex1  i.anlote  c.capune i.hhra  i.mrcms i.anatsi i.hhra c.edhigh1 i.lsdrkf)
putexcel set "C:\Users\mithi\OneDrive - Western Sydney University\HILDA20\MyCODE\Forest Plots\hecpmhp.xlsx", replace
putexcel A1=matrix(r(table))




//////////////////////////////////////////////////////////////////////////////

*Diagnosed with serious illness - Any Serious Mental Illness

stset wave, id(real_xwave) failure(any_mental_illness==1)
 stcox i.num_cluster  c.hgage1 i.hgsex1  i.anlote  , tvc(c.capune i.hhra  i.mrcms i.anatsi i.hhra c.edhigh1 i.lsdrkf   )nolog
 stcoxkm, by(num_cluster) separate
```

```
 *stphplot, by(num_cluster)  adjust(c.hgage1 i.hgsex1  i.anlote  c.capune i.hhra  i.mrcms i.anatsi i.hhra c.edhigh1 i.lsdrkf)
putexcel set "C:\Users\mithi\OneDrive - Western Sydney University\HILDA20\MyCODE\Forest Plots\any_mental_illness.xlsx", replace
putexcel A1=matrix(r(table))

*Takes prescription medication for mental illness  or sees a mental health clinician

//xtpoisson treat_any_mental_illness i.num_cluster  c.hgage1 i.hgsex1  i.anlote  c.capune i.hhra i.mrcms i.hhra c.edhigh1 i.lsdrkf    if any_mental_illness ==1 ,irr difficult


//putexcel set "C:\Users\mithi\OneDrive - Western Sydney University\HILDA20\MyCODE\Forest Plots\treat_any_mental_illness.xlsx", replace
```

```
clear
clear mata
clear matrix

set min_memory 8g
set maxvar 10000

use "C:\Users\mithi\OneDrive - Western Sydney University\HILDA20\FinalData20\ConciseHILDA.dta", replace


xtset real_xwave wave

xtreg bmi i.num_clusters c.hgage1 i.hgsex1 i.hhra c.edhigh1 i.lsdrkf   i.anlote  c.capune

mat list r(table)
 putexcel set "C:\Users\mithi\OneDrive - Western Sydney University\HILDA20\MyCODE\Forest Plots\bmi.xlsx", replace
putexcel A1 = matrix(r(table))

/////////////////////////////////////////////////////////////////////////////////

*Ever been told by a doctor or nurse that you have any of these serious illnesses
stset wave, id(real_xwave) failure(heany==1)
 stcox i.num_cluster  c.hgage1 i.hgsex1  i.anlote  , tvc(c.capune i.hhra  i.mrcms i.anatsi i.hhra c.edhigh1 i.lsdrkf   )

* *stphplot, by(num_cluster)  adjust(c.hgage1 i.hgsex1  i.anlote  c.capune i.hhra  i.mrcms i.anatsi i.hhra c.edhigh1 i.lsdrkf)
stcoxkm, by(num_cluster) separate
mat list r(table)
 putexcel set "C:\Users\mithi\OneDrive - Western Sydney University\HILDA20\MyCODE\Forest Plots\heany.xlsx", replace
putexcel A1 = matrix(r(table))

////////////////////////////////////////////////////////////////////////////////

*   Diagnosed with serious illness - Arthritis or osteoporosis

stset wave, id(real_xwave) failure(heart==1)
 stcox i.num_cluster  c.hgage1 i.hgsex1  i.anlote  , tvc(c.capune i.hhra  i.mrcms i.anatsi i.hhra c.edhigh1 i.lsdrkf   )
stcoxkm, by(num_cluster) separate

*stphplot, by(num_cluster)  adjust(c.hgage1 i.hgsex1  i.anlote  c.capune i.hhra  i.mrcms i.anatsi i.hhra c.edhigh1 i.lsdrkf)
mat list r(table)
 putexcel set "C:\Users\mithi\OneDrive - Western Sydney University\HILDA20\MyCODE\Forest Plots\heart.xlsx", replace
putexcel A1 = matrix(r(table))
*Takes prescription medication for - Arthritis or osteoporosis

xtpoisson hepmart i.num_cluster  c.hgage1 i.hgsex1  i.anlote  c.capune i.hhra  i.mrcms i.anatsi i.hhra c.edhigh1 i.lsdrkf   if heart ==1, irr


mat list r(table)
 putexcel set "C:\Users\mithi\OneDrive - Western Sydney University\HILDA20\MyCODE\Forest Plots\hepmart.xlsx", replace
putexcel A1 = matrix(r(table))
```

```
///////////////////////////////////////////////////////////////////////////

*   Diagnosed with serious illness - Any type of cancer

stset wave, id(real_xwave) failure(hecan==1)
 stcox i.num_cluster  c.hgage1 i.hgsex1  i.anlote  , tvc(c.capune i.hhra  i.mrcms i.anatsi i.hhra c.edhigh1 i.lsdrkf   )
stcoxkm, by(num_cluster) separate

 *stphplot, by(num_cluster)  adjust(c.hgage1 i.hgsex1  i.anlote  c.capune i.hhra  i.mrcms i.anatsi i.hhra c.edhigh1 i.lsdrkf)

mat list r(table)
 putexcel set "C:\Users\mithi\OneDrive - Western Sydney University\HILDA20\MyCODE\Forest Plots\hecan.xlsx", replace
putexcel A1 = matrix(r(table))
*Takes prescription medication for - Any type of cancer

xtpoisson hepmcan i.num_cluster  c.hgage1 i.hgsex1  i.anlote  c.capune i.hhra  i.mrcms i.anatsi i.hhra c.edhigh1 i.lsdrkf   if hecan ==1, irr

mat list r(table)
 putexcel set "C:\Users\mithi\OneDrive - Western Sydney University\HILDA20\MyCODE\Forest Plots\hepmcan.xlsx", replace
putexcel A1 = matrix(r(table))









///////////////////////////////////////////////////////////////////////////

*Diagnosed with serious illness - Chronic bronchitis or emphysema

stset wave, id(real_xwave) failure(hecbe==1)
 stcox i.num_cluster  c.hgage1 i.hgsex1  i.anlote  , tvc(c.capune i.hhra  i.mrcms i.anatsi i.hhra c.edhigh1 i.lsdrkf   )
stcoxkm, by(num_cluster) separate

*stphplot, by(num_cluster)  adjust(c.hgage1 i.hgsex1  i.anlote  c.capune i.hhra  i.mrcms i.anatsi i.hhra c.edhigh1 i.lsdrkf)
mat list r(table)
 putexcel set "C:\Users\mithi\OneDrive - Western Sydney University\HILDA20\MyCODE\Forest Plots\hecbe.xlsx", replace
putexcel A1 = matrix(r(table))
*Takes prescription medication for - Chronic bronchitis or emphysema

xtpoisson hepmcbe i.num_cluster  c.hgage1 i.hgsex1  i.anlote  c.capune i.hhra  i.mrcms i.anatsi i.hhra c.edhigh1 i.lsdrkf   if hecbe ==1, irr

mat list r(table)
 putexcel set "C:\Users\mithi\OneDrive - Western Sydney University\HILDA20\MyCODE\Forest Plots\hepmcbe.xlsx", replace
putexcel A1 = matrix(r(table))

*Diagnosed with serious illness - Asthma

stset wave, id(real_xwave) failure(heast==1)
```

```
 stcox i.num_cluster  c.hgage1 i.hgsex1  i.anlote  , tvc(c.capune i.hhra  i.mrcms i.anatsi i.hhra c.edhigh1 i.lsdrkf i.lsdrkf  )
stcoxkm, by(num_cluster) separate

*stphplot, by(num_cluster)  adjust(c.hgage1 i.hgsex1  i.anlote  c.capune i.hhra  i.mrcms i.anatsi i.hhra c.edhigh1 i.lsdrkf)
mat list r(table)
 putexcel set "C:\Users\mithi\OneDrive - Western Sydney University\HILDA20\MyCODE\Forest Plots\heast.xlsx", replace
putexcel A1 = matrix(r(table))




*Takes prescription medication for - Asthma


xtpoisson hepmast i.num_cluster  c.hgage1 i.hgsex1  i.anlote  c.capune i.hhra  i.mrcms i.anatsi i.hhra c.edhigh1 i.lsdrkf   if heast ==1, irr


mat list r(table)
 putexcel set "C:\Users\mithi\OneDrive - Western Sydney University\HILDA20\MyCODE\Forest Plots\hepmast.xlsx", replace
putexcel A1 = matrix(r(table))






//////////////////////////////////////////////////////////////////////
*   Diagnosed with serious illness - Type 1 diabetes (also known as juvenile-onset or insulin-dependent diabetes==1)

stset wave, id(real_xwave) failure(hedi1==1)
 stcox i.num_cluster  c.hgage1 i.hgsex1  i.anlote  , tvc(c.capune i.hhra  i.mrcms i.anatsi i.hhra c.edhigh1 i.lsdrkf   )
stcoxkm, by(num_cluster) separate

 *stphplot, by(num_cluster)  adjust(c.hgage1 i.hgsex1  i.anlote  c.capune i.hhra  i.mrcms i.anatsi i.hhra c.edhigh1 i.lsdrkf)

mat list r(table)
 putexcel set "C:\Users\mithi\OneDrive - Western Sydney University\HILDA20\MyCODE\Forest Plots\hedi1.xlsx", replace
putexcel A1 = matrix(r(table))
*Takes prescription medication for - Type 1 diabetes (also known as juvenile-onset or insulin-dependent diabetes

xtpoisson hepmdi1 i.num_cluster  c.hgage1 i.hgsex1  i.anlote  c.capune i.hhra  i.mrcms i.anatsi i.hhra c.edhigh1 i.lsdrkf   if hedi1 ==1, irr

mat list r(table)
 putexcel set "C:\Users\mithi\OneDrive - Western Sydney University\HILDA20\MyCODE\Forest Plots\hepmdi1.xlsx", replace
putexcel A1 = matrix(r(table))
*Diagnosed with serious illness - Type 2 diabetes (also known as late-onset or non-insulin dependent diabetes==1)

stset wave, id(real_xwave) failure(hedi2==1)
 stcox i.num_cluster  c.hgage1 i.hgsex1  i.anlote  , tvc(c.capune i.hhra  i.mrcms i.anatsi i.hhra c.edhigh1 i.lsdrkf   )
```

```
stcoxkm, by(num_cluster) separate

 *stphplot, by(num_cluster)  adjust(c.hgage1 i.hgsex1  i.anlote  c.capune i.hhra  i.mrcms i.anatsi i.hhra c.edhigh1 i.lsdrkf)

mat list r(table)
 putexcel set "C:\Users\mithi\OneDrive - Western Sydney University\HILDA20\MyCODE\Forest Plots\hedi2.xlsx", replace
putexcel A1 = matrix(r(table))
*   Takes prescription medication for - Type 2 diabetes (also known as late-onset or non-insulin dependent diabetes==1)

xtpoisson hepmdi2 i.num_cluster  c.hgage1 i.hgsex1  i.anlote  c.capune i.hhra  i.mrcms i.anatsi i.hhra c.edhigh1 i.lsdrkf   if hedi2 ==1, irr

mat list r(table)
 putexcel set "C:\Users\mithi\OneDrive - Western Sydney University\HILDA20\MyCODE\Forest Plots\hepmdi2.xlsx", replace
putexcel A1 = matrix(r(table))










////////////////////////////////////////////////////////////////////////////////
*Diagnosed with serious illness - High blood pressure or hypertension

stset wave, id(real_xwave) failure(hehbp==1)
 stcox i.num_cluster  c.hgage1 i.hgsex1  i.anlote  , tvc(c.capune i.hhra  i.mrcms i.anatsi i.hhra c.edhigh1 i.lsdrkf   )
stcoxkm, by(num_cluster) separate

 *stphplot, by(num_cluster)  adjust(c.hgage1 i.hgsex1  i.anlote  c.capune i.hhra  i.mrcms i.anatsi i.hhra c.edhigh1 i.lsdrkf)

mat list r(table)
 putexcel set "C:\Users\mithi\OneDrive - Western Sydney University\HILDA20\MyCODE\Forest Plots\hehbp.xlsx", replace
putexcel A1 = matrix(r(table))
*Takes prescription medication for - High blood pressure or hypertension

xtpoisson hepmhbp i.num_cluster  c.hgage1 i.hgsex1  i.anlote  c.capune i.hhra  i.mrcms i.anatsi i.hhra c.edhigh1 i.lsdrkf   if hehbp ==1, irr

mat list r(table)
 putexcel set "C:\Users\mithi\OneDrive - Western Sydney University\HILDA20\MyCODE\Forest Plots\hepmhbp.xlsx", replace
putexcel A1 = matrix(r(table))
*   Diagnosed with serious illness - Heart disease

stset wave, id(real_xwave) failure(hehcd==1)
 stcox i.num_cluster  c.hgage1 i.hgsex1  i.anlote  , tvc(c.capune i.hhra  i.mrcms i.anatsi i.hhra c.edhigh1 i.lsdrkf   )
 stcoxkm, by(num_cluster) separate
*stphplot, by(num_cluster)  adjust(c.hgage1 i.hgsex1  i.anlote  c.capune i.hhra  i.mrcms i.anatsi i.hhra c.edhigh1 i.lsdrkf)
stcoxkm, by(num_cluster) separate

```

```
mat list r(table)
 putexcel set "C:\Users\mithi\OneDrive - Western Sydney University\HILDA20\MyCODE\Forest Plots\hehcd.xlsx", replace
putexcel A1 = matrix(r(table))
*Takes prescription medication for - Heart disease

xtpoisson hepmhd i.num_cluster  c.hgage1 i.hgsex1  i.anlote  c.capune i.hhra  i.mrcms i.anatsi i.hhra c.edhigh1 i.lsdrkf   if hehcd ==1, irr

mat list r(table)
 putexcel set "C:\Users\mithi\OneDrive - Western Sydney University\HILDA20\MyCODE\Forest Plots\hepmhd.xlsx", replace
putexcel A1 = matrix(r(table))
*   Diagnosed with serious illness - Any other serious circulatory condition (eg stroke, hardening of the arteries==1)

stset wave, id(real_xwave) failure(heoc==1)
 stcox i.num_cluster  c.hgage1 i.hgsex1  i.anlote  , tvc(c.capune i.hhra  i.mrcms i.anatsi i.hhra c.edhigh1 i.lsdrkf   )
*stphplot, by(num_cluster)  adjust(c.hgage1 i.hgsex1  i.anlote  c.capune i.hhra  i.mrcms i.anatsi i.hhra c.edhigh1 i.lsdrkf)
stcoxkm, by(num_cluster) separate

mat list r(table)
 putexcel set "C:\Users\mithi\OneDrive - Western Sydney University\HILDA20\MyCODE\Forest Plots\heoc.xlsx", replace
putexcel A1 = matrix(r(table))
*Takes prescription medication for - Any other serious circulatory condition (eg stroke, hardening of the arteries==1)

*xtpoisson hepmoc i.num_cluster  c.hgage1 i.hgsex1  i.anlote  c.capune i.hhra  i.mrcms i.anatsi i.hhra c.edhigh1 i.lsdrkf   if heoc ==1, irr

*mat list r(table)
* putexcel set "C:\Users\mithi\OneDrive - Western Sydney University\HILDA20\MyCODE\Forest Plots\hepmoc.xlsx", replace
*putexcel A1 = matrix(r(table))



////////////////////////////////////////////////////////////////////
*Any cardiovascular disease

stset wave, id(real_xwave) failure(htn_hd_pvd_stroke==1)
 stcox i.num_cluster  c.hgage1 i.hgsex1  i.anlote  , tvc(c.capune i.hhra  i.mrcms i.anatsi i.hhra c.edhigh1 i.lsdrkf   )
 stcoxkm, by(num_cluster) separate

*stphplot, by(num_cluster)  adjust(c.hgage1 i.hgsex1  i.anlote  c.capune i.hhra  i.mrcms i.anatsi i.hhra c.edhigh1 i.lsdrkf)
mat list r(table)
 putexcel set "C:\Users\mithi\OneDrive - Western Sydney University\HILDA20\MyCODE\Forest Plots\htn_hd_pvd_stroke.xlsx", replace
putexcel A1 = matrix(r(table))

//Takes prescription medication for - Any cardiovascular diseases


xtpoisson treat_htn_hd_pvd_stroke i.num_cluster  c.hgage1 i.hgsex1  i.anlote  c.capune i.hhra  i.mrcms i.anatsi i.hhra c.edhigh1 i.lsdrkf   if htn_hd_pvd_stroke ==1, irr

mat list r(table)
 putexcel set "C:\Users\mithi\OneDrive - Western Sydney University\HILDA20\MyCODE\Forest
```

```
Plots\treat_htn_hd_pvd_stroke.xlsx", replace
putexcel A1 = matrix(r(table))
```

```
use "C:\Users\mithi\OneDrive - Western Sydney University\HILDA20\FinalData20\ConciseHILDA.dta", replace



//mdesc  hgage1 hgsex1 gh1 gh10 edhigh1 mrcms capune pdk10s anlote lsdrkf hhra   lejlf levio  lebth dodtyp bmi  anatsi if num_cluster!=. , any

//mdesc  hgage1 hgsex1 gh1 gh10 edhigh1 mrcms capune pdk10s anlote lsdrkf hhra   lejlf levio  lebth dodtyp bmi  anatsi if num_cluster!=.


//mlogit num_clusters  hgage1 hgsex1 gh1 gh10 edhigh1 mrcms capune pdk10s anlote lsdrkf hhra   lejlf levio  lebth dodtyp bmi  anatsi , rrr
cls

mlogit num_clusters  hgage1 i.hgsex1 i.gh1 gh10 c.edhigh1 i.mrcms c.capune c.pdk10s i.anlote i.lsdrkf i.hhra   i.lejlf i.levio  i.lebth ib3.dodtyp c.bmi  i.anatsi , rrr baseoutcome(1)

drop if hgage1==. | hgsex1 ==. | gh1 ==. | gh10 ==. | edhigh1 ==. | mrcms ==. | capune ==. | pdk10s ==. | anlote ==. | lsdrkf ==. | hhra ==. | lejlf ==. | levio ==. |  lebth ==. | dodtyp ==. | bmi ==. | anatsi ==. | num_clusters ==.



quietly tabulate mrcms, generate(new_mrcms_)

quietly tabulate lsdrkf, generate(new_lsdrkf)

quietly tabulate dodtyp, generate(new_dodtyp)

quietly tabulate anatsi, generate(new_anatsi)

recode anlote (2=0)

recode hgsex1 (2=0)

replace lejlf = lejlf -1


replace levio = levio -1

replace lebth = lebth -1

drop new_dodtyp16 new_dodtyp15 new_dodtyp14 new_dodtyp13 new_dodtyp12 new_dodtyp11 new_dodtyp10 new_dodtyp9 new_dodtyp8 new_dodtyp7 new_dodtyp6 new_dodtyp5 new_dodtyp4 new_dodtyp2 new_dodtyp1 dodtyp heany heart hecan hecbe hedi1 hedi2 hedep heomi hehcd hehbp heoc hepmart hepmast hepmcan hepmcbe hepmdi1 hepmdi2 hepmdep hepmomi hepmhd hepmhbp hepmoc hecpmhp treat_any_mental_illness any_mental_illness treat_htn_hd_pvd_stroke htn_hd_pvd_stroke negwave

recode new_anatsi1 (0=1) (1=0)

rename new_anatsi1 ATSI


drop real_xwaveid wave hhrhid new_anatsi2 new_anatsi3 new_anatsi4  mrcms
label variable ATSI "anatsi==[1] Aboriginal and or Torres Strait Islander"

export delimited using "C:\Users\mithi\OneDrive - Western Sydney University\HILDA20\ml\HILDAminPython.csv", replace


save "C:\Users\mithi\OneDrive - Western Sydney University\HILDA20\ml\HILDAml.dta", replace
```

```
use "C:\Users\mithi\OneDrive - Western Sydney University\HILDA20\ml\HILDAml.dta", replace

cd "C:\Users\mithi\OneDrive - Western Sydney University\HILDA20\ml\"



/*


//RANDOM FOREST - dont need cross validation
 splitsample, generate(svar, replace) split(0.80 0.20)

    preserve
            keep if svar==1
            save HILDA_train , replace
            restore
  //      Form the test dataset
            preserve
            keep if svar==2
            save HILDA_test , replace
            restore
    //    Load train dataset
            use HILDA_train, clear
      //  Run tree regression
c_ml_stata_cv num_clusters  hgage1 hgsex1 gh1 gh10 edhigh1 new_mrcms_2 new_mrcms_3  new_mrcms_4 new_mrcms_5 new_mrcms_6 capune pdk10s anlote new_lsdrkf2 new_lsdrkf3 new_lsdrkf4 new_lsdrkf5 new_lsdrkf6 new_lsdrkf7 new_lsdrkf8 hhra   lejlf levio  lebth  new_dodtyp1  new_dodtyp2 new_dodtyp4  new_dodtyp5  new_dodtyp6  new_dodtyp7  new_dodtyp8  new_dodtyp9  new_dodtyp10 new_dodtyp11  new_dodtyp12  new_dodtyp13  new_dodtyp14  new_dodtyp15 bmi new_anatsi2  new_anatsi3 new_anatsi4  , mlmodel("randomforest") data_test("HILDA_test")  default prediction("pred") seed(1) graph_cv save_graph_cv(testing)



    mat coef1 =     e(OPT_N_ESTIMATORS)
    mat coef2 =   e(OPT_TREE_DEPTH)
      mat coef3 = e(OPT_MAX_FEATURES)
  mat coef4 =     e(N_train_all)
      mat coef5 = e(N_train_used)
      mat coef6 = e(N_test_all)
mat coef7 =       e(N_test_used)
mat coef8 =       e(N_features)
mat coef9 =       e(TRAIN_ACCURACY)
mat coef10 =       e(TEST_ACCURACY)
mat coef11 =       e(SE_TEST_ACCURACY)
mat coef12 =       e(BEST_INDEX)
mat coef13 =       e(N_FOLDS)
mat coef14 =       e(Train_mse)
mat coef15 =       e(Test_mse)
mat coef16 =       e(Train_mape)
mat coef17 =       e(Test_mape)

mat li coef1
mat li coef2
mat li coef3
mat li coef4
mat li coef5
mat li coef6
mat li coef7
mat li coef8

mat li coef9
```

```
mat li coef10
mat li coef11
mat li coef12
mat li coef13
mat li coef14
mat li coef15
mat li coef16
mat li coef17
```

```
#install.packages("ggpubr")
#install.packages("qpdf")
#################################MIND######################

library(readxl)
library(ggplot2)
library(ggpubr)



Misc = list()
Lung = list()
CVD = list()
Diab = list()
Psych = list()



misc<-c('heany','hecan','hepmcan','heart','hepmart')

Lung_Disease<-c('hecbe','hepmcbe','heast','hepmast')

Diabetes<-c('hedi1','hepmdi1','hedi2','hepmdi2')

Cardiovascular_Disease<-
c('hehbp','hepmhbp','hehcd','hepmhd','heoc','htn_hd_pvd_stroke','treat_htn_hd_pvd_stroke')

Mind_Outcomes<-c('hedep','hepmdep','heomi','hecpmhp','Changes in K10(Distress)
over time','any_mental_illness', 'treat_any_mental_illness')




for (outcome in misc){


  myCols <- c("Upper Middle Class","Lower Middle Class","Low
Income","Risers","Fallers")
  myRows <- c("RR","SE","Z","P-Value","L95CI","U95CI")

  ExcelFile<-paste0("C:/Users/mithi/OneDrive - Western Sydney University/
HILDA20/MyCODE/Forest Plots/",outcome,".xlsx", sep="")

  outcome1<-as.matrix(read_excel(ExcelFile, range = "B1:F6",col_names =
myCols))
  rownames(outcome1) <- myRows

  outcome1<-t(outcome1)

  outcome1<-data.frame(outcome1)

  outcome1$Clusters6 <-myCols

  rownames(outcome1)<- NULL
```

```
  outcome1$RR <- as.numeric(outcome1$RR)
  outcome1$L95CI <- as.numeric(outcome1$L95CI)
  outcome1$U95CI <- as.numeric(outcome1$U95CI)
  outcome1$index <-as.numeric(c(1:5))


  #create data
  df <- data.frame(study=c("Decreasing Income (UMI to LMI)","Increasing Income
(LMI to UMI)","Low Income","Low Middle Income","Upper Middle Income"),
                   index=5:1,
                   effect=outcome1$RR,
                   lower=outcome1$L95CI,
                   upper=outcome1$U95CI)

  df<-rbind(df, data.frame(study="High
Income",index=6,effect=1,lower=1,upper=1  ))


  outcome1_plot <- ggplot(data=df, aes(y=index, x=effect, xmin=lower,
xmax=upper)) +
    geom_point() +
    geom_errorbarh(height=.1) +
    scale_y_continuous(breaks=1:nrow(df), labels=df$study) +
    scale_x_continuous(limits=c(0.5,4),trans="log2",breaks=c(0.5,1,2,4)) +
    labs(title=outcome, x='Hazard Ratio', y = '') +
    geom_vline(xintercept=1, color='black', linetype='dashed', alpha=.5) +
    theme_classic()



  Misc[[outcome]] = outcome1_plot

}


##########################################################################################


for (outcome in Lung_Disease){


  myCols <- c("Upper Middle Class","Lower Middle Class","Low
Income","Risers","Fallers")
  myRows <- c("RR","SE","Z","P-Value","L95CI","U95CI")

  ExcelFile<-paste0("C:/Users/mithi/OneDrive - Western Sydney University/
HILDA20/MyCODE/Forest Plots/",outcome,".xlsx", sep="")

  outcome1<-as.matrix(read_excel(ExcelFile, range = "B1:F6",col_names =
myCols))
  rownames(outcome1) <- myRows
```

```
  outcome1<-t(outcome1)

  outcome1<-data.frame(outcome1)

  outcome1$Clusters6 <-myCols

  rownames(outcome1)<- NULL

  outcome1$RR <- as.numeric(outcome1$RR)
  outcome1$L95CI <- as.numeric(outcome1$L95CI)
  outcome1$U95CI <- as.numeric(outcome1$U95CI)
  outcome1$index <-as.numeric(c(1:5))


  #create data
  df <- data.frame(study=c("Decreasing Income (UMI to LMI)","Increasing Income
(LMI to UMI)","Low Income","Low Middle Income","Upper Middle Income"),
                   index=5:1,
                   effect=outcome1$RR,
                   lower=outcome1$L95CI,
                   upper=outcome1$U95CI)

  df<-rbind(df, data.frame(study="High
Income",index=6,effect=1,lower=1,upper=1  ))



  outcome1_plot <- ggplot(data=df, aes(y=index, x=effect, xmin=lower,
xmax=upper)) +
    geom_point() +
    geom_errorbarh(height=.1) +
    scale_y_continuous(breaks=1:nrow(df), labels=df$study) +
    scale_x_continuous(limits=c(0.5,4),trans="log2",breaks=c(0.5,1,2,4)) +
    labs(title=outcome, x='Hazard Ratio', y = '') +
    geom_vline(xintercept=1, color='black', linetype='dashed', alpha=.5) +
    theme_classic()



  Lung[[outcome]] = outcome1_plot

}



####################################################################################################
#Diabetes



for (outcome in Diabetes){
```

```
  myCols <- c("Upper Middle Class","Lower Middle Class","Low
Income","Risers","Fallers")
  myRows <- c("RR","SE","Z","P-Value","L95CI","U95CI")

  ExcelFile<-paste0("C:/Users/mithi/OneDrive - Western Sydney University/
HILDA20/MyCODE/Forest Plots/",outcome,".xlsx", sep="")

  outcome1<-as.matrix(read_excel(ExcelFile, range = "B1:F6",col_names =
myCols))
  rownames(outcome1) <- myRows

  outcome1<-t(outcome1)

  outcome1<-data.frame(outcome1)

  outcome1$Clusters6 <-myCols

  rownames(outcome1)<- NULL

  outcome1$RR <- as.numeric(outcome1$RR)
  outcome1$L95CI <- as.numeric(outcome1$L95CI)
  outcome1$U95CI <- as.numeric(outcome1$U95CI)
  outcome1$index <-as.numeric(c(1:5))


  #create data
  df <- data.frame(study=c("Decreasing Income (UMI to LMI)","Increasing Income
(LMI to UMI)","Low Income","Low Middle Income","Upper Middle Income"),
                   index=5:1,
                   effect=outcome1$RR,
                   lower=outcome1$L95CI,
                   upper=outcome1$U95CI)

  df<-rbind(df, data.frame(study="High
Income",index=6,effect=1,lower=1,upper=1  ))


  outcome1_plot <- ggplot(data=df, aes(y=index, x=effect, xmin=lower,
xmax=upper)) +
    geom_point() +
    geom_errorbarh(height=.1) +
    scale_y_continuous(breaks=1:nrow(df), labels=df$study) +
    scale_x_continuous(limits=c(0.5,4),trans="log2",breaks=c(0.5,1,2,4)) +
    labs(title=outcome, x='Hazard Ratio', y = '') +
    geom_vline(xintercept=1, color='black', linetype='dashed', alpha=.5) +
    theme_classic()



  Diab[[outcome]] = outcome1_plot


}
```

```
##########################################################################################
#Cardiovascular_Disease




for (outcome in Cardiovascular_Disease){


  myCols <- c("Upper Middle Class","Lower Middle Class","Low
Income","Risers","Fallers")
  myRows <- c("RR","SE","Z","P-Value","L95CI","U95CI")

  ExcelFile<-paste0("C:/Users/mithi/OneDrive - Western Sydney University/
HILDA20/MyCODE/Forest Plots/",outcome,".xlsx", sep="")

  outcome1<-as.matrix(read_excel(ExcelFile, range = "B1:F6",col_names =
myCols))
  rownames(outcome1) <- myRows

  outcome1<-t(outcome1)

  outcome1<-data.frame(outcome1)

  outcome1$Clusters6 <-myCols

  rownames(outcome1)<- NULL

  outcome1$RR <- as.numeric(outcome1$RR)
  outcome1$L95CI <- as.numeric(outcome1$L95CI)
  outcome1$U95CI <- as.numeric(outcome1$U95CI)
  outcome1$index <-as.numeric(c(1:5))


  #create data
  df <- data.frame(study=c("Decreasing Income (UMI to LMI)","Increasing Income
(LMI to UMI)","Low Income","Low Middle Income","Upper Middle Income"),
                   index=5:1,
                   effect=outcome1$RR,
                   lower=outcome1$L95CI,
                   upper=outcome1$U95CI)

  df<-rbind(df, data.frame(study="High
Income",index=6,effect=1,lower=1,upper=1  ))


  outcome1_plot <- ggplot(data=df, aes(y=index, x=effect, xmin=lower,
xmax=upper)) +
```

```
    geom_point() +
    geom_errorbarh(height=.1) +
    scale_y_continuous(breaks=1:nrow(df), labels=df$study) +
    scale_x_continuous(limits=c(0.5,4),trans="log2",breaks=c(0.5,1,2,4)) +
    labs(title=outcome, x='Hazard Ratio', y = '') +
    geom_vline(xintercept=1, color='black', linetype='dashed', alpha=.5) +
    theme_classic()



  CVD[[outcome]] = outcome1_plot





}

#################################MIND######################

library(readxl)


library(ggplot2)
library(ggpubr)








for (outcome in Mind_Outcomes){

  myCols <- c("Upper Middle Class","Lower Middle Class","Low
Income","Risers","Fallers")
  myRows <- c("RR","SE","Z","P-Value","L95CI","U95CI")

  ExcelFile<-paste0("C:/Users/mithi/OneDrive - Western Sydney University/
HILDA20/MyCODE/Forest Plots/",outcome,".xlsx", sep="")

  outcome1<-as.matrix(read_excel(ExcelFile, range = "B1:F6",col_names =
myCols))
  rownames(outcome1) <- myRows

  outcome1<-t(outcome1)

  outcome1<-data.frame(outcome1)

  outcome1$Clusters6 <-myCols

  rownames(outcome1)<- NULL

  outcome1$RR <- as.numeric(outcome1$RR)
  outcome1$L95CI <- as.numeric(outcome1$L95CI)
```

```
  outcome1$U95CI <- as.numeric(outcome1$U95CI)
  outcome1$index <-as.numeric(c(1:5))


  #create data
  df <- data.frame(study=c("Decreasing Income (UMI to LMI)","Increasing Income
(LMI to UMI)","Low Income","Low Middle Income","Upper Middle Income"),
                   index=5:1,
                   effect=outcome1$RR,
                   lower=outcome1$L95CI,
                   upper=outcome1$U95CI)

  df<-rbind(df, data.frame(study="High
Income",index=6,effect=1,lower=1,upper=1  ))


  outcome1_plot <- ggplot(data=df, aes(y=index, x=effect, xmin=lower,
xmax=upper)) +
    geom_point() +
    geom_errorbarh(height=.1) +
    scale_y_continuous(breaks=1:nrow(df), labels=df$study) +
    scale_x_continuous(limits=c(0.5,4),trans="log2",breaks=c(0.5,1,2,4)) +
    labs(title=outcome, x='Hazard Ratio', y = '') +
    geom_vline(xintercept=1, color='black', linetype='dashed', alpha=.5) +
    theme_classic()



  Psych[[outcome]] = outcome1_plot






}

##################################333
#Generating Graphics

TiffFile="C:/Users/mithi/OneDrive - Western Sydney University/HILDA20/Results/
MindFP.tiff"
tiff(TiffFile, units="in", width=14, height=9, res=300)

Psych$hedep$labels$title <-"New Onset Depression/Anxiety"
Psych$hepmdep$labels$title <-"Commenced Treatment  for Depression or Anxiety"
Psych$heomi$labels$title <-"New Onset for Mental Illness (Not Depression/
Anxiety)"

Psych$any_mental_illness$labels$title <-"New Onset for Any Serious Mental
Illness"
Psych$treat_any_mental_illness$labels$title <-"Commenced Treatment for Serious
Mental Illness"
Psych$hecpmhp$labels$title <-"First visit to Mental Health Clinician"
```

```
Psych$round_k10s_ipol$title <- "Changes in K10 (Distress) over time"

Psych$New = NA

print(ggarrange(Psych$hedep , Psych$hepmdep  ,
                Psych$heomi , Psych$`Changes in K10(Distress) over time`,
                Psych$hecpmhp , Psych$New,Psych$any_mental_illness,
Psych$treat_any_mental_illness,
                ncol=2,
                nrow=3))
dev.off()




TiffFile="C:/Users/mithi/OneDrive - Western Sydney University/HILDA20/Results/
Body_DiabetesFP.tiff"
tiff(TiffFile, units="in", width=14, height=6, res=300)


Diab$hedi1$labels$title <-"New Onset T1 Diabetes (New)"
Diab$hepmdi1$labels$title <-"Commenced Treatment for T1 Diabetes"
Diab$hedi2$labels$title <-"New Onset T2 Diabetes"
Diab$hepmdi2$labels$title <-"Commenced Treatment for T2 Diabetes"


print(ggarrange(Diab$hedi1 ,
                Diab$hepmdi1  ,
                Diab$hedi2 ,
                Diab$hepmdi2 ,
                ncol=2,
                nrow=2))
dev.off()


TiffFile="C:/Users/mithi/OneDrive - Western Sydney University/HILDA20/Results/
Body_MiscFP.tiff"
tiff(TiffFile, units="in", width=14, height=9, res=300)

Misc$heany$labels$title <-"New Onset Serious Illness"
Misc$hecan$labels$title <-"New Onset Cancer"
Misc$hepmcan$labels$title <-"Commenced Treatment for Cancer"
Misc$heart$labels$title <-"New Onset Arthritis / Osteoporosis"
Misc$hepmart$labels$title <-"Commenced Treatment for Arthritis / Osteoporosis"
Misc$new=NA
print(ggarrange(Misc$heany ,Misc$new,
                Misc$hecan ,
                Misc$hepmcan ,
                Misc$heart ,
                Misc$hepmart ,
                ncol=2,
                nrow=3))
dev.off()
```

```
TiffFile="C:/Users/mithi/OneDrive - Western Sydney University/HILDA20/Results/
Body_LungFP.tiff"
tiff(TiffFile, units="in", width=14, height=6, res=300)

Lung$hecbe$labels$title <-"New Onset Chronic Bronchitis or Emphysema"
Lung$hepmcbe$labels$title <-"Commenced Treatment for Chronic Bronchitis or
Emphysema"
Lung$heast$labels$title <-"New Onset Asthma"
Lung$hepmast$labels$title <-"Commenced Treatment for Asthma"
Lung$New = NA
print(ggarrange(Lung$hecbe ,
                Lung$hepmcbe  , Lung$heast,
                Lung$hepmast ,
                ncol=2,
                nrow=2))
dev.off()


TiffFile="C:/Users/mithi/OneDrive - Western Sydney University/HILDA20/Results/
Body_CardiovascularFP.tiff"
tiff(TiffFile, units="in", width=14, height=9, res=300)
#CHECK_CARDIAC


CVD$hehbp$labels$title <-"New Onset Hypertension"
CVD$hepmhbp$labels$title <-"Commenced Treatment for Hypertension"
CVD$hehcd$labels$title <-"New Onset Heart Disease"
CVD$hepmhd$labels$title <-"Commenced Treatment for Heart Disease"
CVD$heoc$labels$title <-"New Onset Preipheral Vascular Disease & Stroke"
CVD$hepmoc$labels$title <-"Commenced Treatment for Preipheral Vascular Disease
& Stroke"
CVD$htn_hd_pvd_stroke$labels$title <-"Any New Onset CVD or PVD or
Hypertension"
CVD$treat_htn_hd_pvd_stroke$labels$title <-"Commenced Treatment for Any CVD or
PVD or Hypertension"



print(
  ggarrange(CVD$hehbp ,
            CVD$hepmhbp   ,
            CVD$hehcd ,
            CVD$hepmhd ,
            CVD$heoc ,
            CVD$hepmoc  ,
            CVD$htn_hd_pvd_stroke,
            CVD$treat_htn_hd_pvd_stroke,
            ncol=2,
            nrow=4)

)
dev.off()
```

```
##################################
#Generating PDFs

PDFFileMind="C:/Users/mithi/OneDrive - Western Sydney University/HILDA20/
Results/MindFP.pdf"
pdf(PDFFileMind, width=13.33, height=7.5)


Psych$hedep$labels$title <-"New Onset Depression/Anxiety"
Psych$hepmdep$labels$title <-"Commenced Treatment  for Depression or Anxiety"
Psych$heomi$labels$title <-"New Onset for Mental Illness (Not Depression/
Anxiety)"
Psych$any_mental_illness$labels$title <-"New Onset for Any Serious Mental
Illness"
Psych$treat_any_mental_illness$labels$title <-"Commenced Treatment for Serious
Mental Illness"
Psych$hecpmhp$labels$title <-"First visit to Mental Health Clinician"
Psych$round_k10s_ipol$title <- "Changes in K10 (Distress) over time"

Psych$New = NA

print(ggarrange(Psych$hedep , Psych$hepmdep  ,
                Psych$heomi  , Psych$`Changes in K10(Distress) over time`,
                Psych$hecpmhp , Psych$New,
Psych$any_mental_illness,Psych$treat_any_mental_illness ,
                ncol=2,
                nrow=4))
dev.off()




PDFFileDiab="C:/Users/mithi/OneDrive - Western Sydney University/HILDA20/
Results/Body_DiabetesFP.pdf"
pdf(PDFFileDiab,  width=13.33, height=7.5)


Diab$hedi1$labels$title <-"New Onset T1 Diabetes (New)"
Diab$hepmdi1$labels$title <-"Commenced Treatment for T1 Diabetes"
Diab$hedi2$labels$title <-"New Onset T2 Diabetes"
Diab$hepmdi2$labels$title <-"Commenced Treatment for T2 Diabetes"


print(ggarrange(Diab$hedi1 ,
                Diab$hepmdi1  ,
                Diab$hedi2 ,
                Diab$hepmdi2 ,
                ncol=2,
                nrow=2))
dev.off()
```

```
PDFFileMisc="C:/Users/mithi/OneDrive - Western Sydney University/HILDA20/
Results/Body_MiscFP.pdf"
pdf(PDFFileMisc,  width=13.33, height=7.5)

Misc$heany$labels$title <-"New Onset Serious Illness"
Misc$hecan$labels$title <-"New Onset Cancer"
Misc$hepmcan$labels$title <-"Commenced Treatment for Cancer"
Misc$heart$labels$title <-"New Onset Arthritis / Osteoporosis"
Misc$hepmart$labels$title <-"Commenced Treatment for Arthritis / Osteoporosis"
Misc$new=NA
print(ggarrange(Misc$heany ,Misc$new,
                Misc$hecan ,
                Misc$hepmcan ,
                Misc$heart ,
                Misc$hepmart ,
                ncol=2,
                nrow=3))
dev.off()


PDFFileLung="C:/Users/mithi/OneDrive - Western Sydney University/HILDA20/
Results/Body_LungFP.pdf"
pdf(PDFFileLung,  width=13.33, height=7.5)

Lung$hecbe$labels$title <-"New Onset Chronic Bronchitis or Emphysema"
Lung$hepmcbe$labels$title <-"Commenced Treatment for Chronic Bronchitis or
Emphysema"
Lung$hepmast$labels$title <-"Commenced Treatment for Asthma"
Lung$New = NA
print(ggarrange(Lung$hecbe ,
                Lung$hepmcbe  , Lung$heast,
                Lung$hepmast ,
                ncol=2,
                nrow=2))
dev.off()


PDFFileCardiac="C:/Users/mithi/OneDrive - Western Sydney University/HILDA20/
Results/Body_CardiovascularFP.pdf"
pdf(PDFFileCardiac,  width=13.33, height=7.5)
#CHECK_CARDIAC


CVD$hehbp$labels$title <-"New Onset Hypertension"
CVD$hepmhbp$labels$title <-"Commenced Treatment for Hypertension"
CVD$hehcd$labels$title <-"New Onset Heart Disease"
CVD$hepmhd$labels$title <-"Commenced Treatment for Heart Disease"
CVD$heoc$labels$title <-"New Onset Preipheral Vascular Disease & Stroke"
CVD$hepmoc$labels$title <-"Commenced Treatment for Preipheral Vascular Disease
& Stroke"
CVD$htn_hd_pvd_stroke$labels$title <-"Any New Onset CVD or PVD or
Hypertension"
CVD$treat_htn_hd_pvd_stroke$labels$title <-"Commenced Treatment for CVD or PVD
or Hypertension"
```

```
print(
  ggarrange(CVD$hehbp ,
            CVD$hepmhbp   ,
            CVD$hehcd ,
            CVD$hepmhd ,
            CVD$heoc ,
            CVD$hepmoc  ,
            CVD$htn_hd_pvd_stroke,
            CVD$treat_htn_hd_pvd_stroke,
            ncol=2,
            nrow=4)

)
dev.off()

SixClusters="C:/Users/mithi/OneDrive - Western Sydney University/HILDA20/
Results/6clusters_HILDA20.pdf"
Title1="C:/Users/mithi/OneDrive - Western Sydney University/HILDA20/Results/
Title.pdf"
Final1 = "C:/Users/mithi/OneDrive - Western Sydney University/HILDA20/Results/
FinalResults/FinalHILDA20Graphs.pdf"
qpdf::pdf_combine(input = c(Title1, SixClusters, PDFFileMind, PDFFileMisc,
PDFFileCardiac,  PDFFileDiab,PDFFileLung),
                  output = Final1)
```

```
rm(list = ls())
library(readxl)
library(tidyverse)
setwd("C:/Users/mithi/OneDrive - Western Sydney University/HILDA20/Results/
UnifiedForestPlot")
dados <- data.frame(read_excel("C:/Users/mithi/OneDrive - Western Sydney
University/HILDA20/Results/UnifiedForestPlot/SocMob_outputv4.xlsx"))

dados_ph = dados %>%
    filter(Included_Ilness %in% 1)


# Another option is to use forest from metafor package
library(metafor)

dados_ph$LCI <- log(dados_ph$LCI)
dados_ph$UCI <- log(dados_ph$UCI)
dados_ph$RR <- log(dados_ph$RR)
tiff("C:/Users/mithi/OneDrive - Western Sydney University/HILDA20/Results/
UnifiedForestPlot/SeriousIllness.tif", width = 45, height = 75, units = "cm",
pointsize = 12,
     bg = "white", res = 300, family = "", compression="lzw")
forest(x = dados_ph$RR,efac=c(0,1),
       xlim = c(-3,2.5),
       alim = c(-0.693147181,1.386294361),atransf = exp,
       ci.lb = dados_ph$LCI,
       ci.ub = dados_ph$UCI,
       xlab = "Relative Risk",
       header = c(NA,"RR(95%CI)"),  cex = 1.50,
       slab = NA,  psize = 1.25,at = c(-1.386294361,
                        -0.693147181,-0.287682072,
                        0,
                        0.405465108,
                        0.693147181,
                        1.386294361) ,
       rows = c(105:100,96:91,87:82,78:73,69:64,60:55,51:46,42:37,33:28,
24:19, 15:10, 6:1 ),
            main = "Risk of Health Outcomes and New Onset Serious Illness by
Socio-economic Position and Social Mobility (SEP)", cex.main = 2.0)

abline(h=8,v=0)
abline(h=17,v=0)
abline(h=26,v=0)
abline(h=35,v=0)
abline(h=44,v=0)
abline(h=53,v=0)
abline(h=62,v=0)
abline(h=71,v=0 )
abline(h=80,v=0)
abline(h=89,v=0)
abline(h=98,v=0)
```

```
text(-1.25, c(107,105,96,87,78,69,60,51,42,33,24,15,6),cex = 1.75 , pos = 2,
font = 2, c("Health Outcomes/New Onset Illness","Rise in Distress(K10)","Rise
in Obesity(BMI)","Any Serious Illness","Depression or Anxiety","Type 1
Diabetes","
Type 2 Diabetes","Hypertension","Heart Disease","Chronic Bronchitis or
Emphysema","Asthma","Any Cancer Diagnosis","
Arthritis or Osteoporosis"))

text(-0.75, c(107),cex = 1.75 , pos = 2, font = 2, c("SEP"))

text(-0.75, c(105:100),cex = 1.50 , pos = 2, font = 0.5, c("High","Upper-
mid","Lower-mid","Low","Increasing","Decreasing"))
text(-0.75, c(96:91),cex = 1.50 , pos = 2, font = 0.5, c("High","Upper-
mid","Lower-mid","Low","Increasing","Decreasing"))
text(-0.75, c(87:82),cex = 1.50 , pos = 2, font = 0.5, c("High","Upper-
mid","Lower-mid","Low","Increasing","Decreasing"))
text(-0.75, c(78:73),cex = 1.50 , pos = 2, font = 0.5, c("High","Upper-
mid","Lower-mid","Low","Increasing","Decreasing"))
text(-0.75, c(69:64),cex = 1.50 , pos = 2, font = 0.5, c("High","Upper-
mid","Lower-mid","Low","Increasing","Decreasing"))
text(-0.75, c(60:55),cex = 1.50 , pos = 2, font = 0.5, c("High","Upper-
mid","Lower-mid","Low","Increasing","Decreasing"))
text(-0.75, c(51:46),cex = 1.50 , pos = 2, font = 0.5, c("High","Upper-
mid","Lower-mid","Low","Increasing","Decreasing"))
text(-0.75, c(42:37),cex = 1.50 , pos = 2, font = 0.5, c("High","Upper-
mid","Lower-mid","Low","Increasing","Decreasing"))
text(-0.75, c(33:28),cex = 1.50 , pos = 2, font = 0.5, c("High","Upper-
mid","Lower-mid","Low","Increasing","Decreasing"))
text(-0.75, c(24:19),cex = 1.50 , pos = 2, font = 0.5, c("High","Upper-
mid","Lower-mid","Low","Increasing","Decreasing"))
text(-0.75, c(15:10),cex = 1.50 , pos = 2, font = 0.5, c("High","Upper-
mid","Lower-mid","Low","Increasing","Decreasing"))
text(-0.75, c(6:1),cex = 1.50 , pos = 2, font = 0.5, c("High","Upper-
mid","Lower-mid","Low","Increasing","Decreasing"))


dev.off()
# This has many other options, but it is everything done "by hand"
```

```
rm(list = ls())
library(readxl)
library(tidyverse)
setwd("C:/Users/mithi/OneDrive - Western Sydney University/HILDA20/Results/
UnifiedForestPlot")
dados <- data.frame(read_excel("C:/Users/mithi/OneDrive - Western Sydney
University/HILDA20/Results/UnifiedForestPlot/SocMob_outputv4.xlsx"))

dados_ph = dados %>%
    filter(Included_Ilness %in% 2)


# Another option is to use forest from metafor package
library(metafor)

dados_ph$LCI <- log(dados_ph$LCI)
dados_ph$UCI <- log(dados_ph$UCI)
dados_ph$RR <- log(dados_ph$RR)
tiff("C:/Users/mithi/OneDrive - Western Sydney University/HILDA20/Results/
UnifiedForestPlot/TreatmentRates.tif", width = 40, height = 75, units = "cm",
pointsize = 12,
     bg = "white", res = 300, family = "", compression="lzw")
forest(x = dados_ph$RR,efac=c(0,1),
       xlim = c(-3,2),
       alim = c(-0.693147181,1.386294361),atransf = exp,
       ci.lb = dados_ph$LCI,
       ci.ub = dados_ph$UCI,
       xlab = "Relative Risk",
       header = c(NA,"RR(95%CI)"),  cex = 1.50,
       slab = NA,  psize = 1.25,at = c(-1.386294361,
                        -0.693147181,-0.287682072,
                        0,
                        0.405465108,
                        0.693147181,
                        1.386294361) ,
       rows = c(87:82,78:73,69:64,60:55,51:46,42:37,33:28, 24:19, 15:10, 6:1
),
            main = "Initiation of Treatment for New Onset Serious Illness by
Socio-economic Position and Social Mobility (SEP)", cex.main=1.75)

abline(h=8,v=0)
abline(h=17,v=0)
abline(h=26,v=0)
abline(h=35,v=0)
abline(h=44,v=0)
abline(h=53,v=0)
abline(h=62,v=0)
abline(h=71,v=0 )
abline(h=80,v=0)
```

```
text(-1.25, c(89,87,78,69,60,51,42,33,24,15,6),cex = 1.75 , pos = 2, font = 2, c("Treatment Intitiation Rates","First Visit to a Mental Health Clinician","Depression or Anxiety","Type 1 Diabetes","Type 2 Diabetes","Hypertension","Heart Disease","Chronic Bronchitis or Emphysema","Asthma","Cancer","Arthritis/Osteoporosis"))

text(-0.75, c(89),cex = 1.75 , pos = 2, font = 2, c("SEP"))

text(-0.75, c(87:82),cex = 1.50 , pos = 2, font = 0.5, c("High","Upper-mid","Lower-mid","Low","Increasing","Decreasing"))
text(-0.75, c(78:73),cex = 1.50 , pos = 2, font = 0.5, c("High","Upper-mid","Lower-mid","Low","Increasing","Decreasing"))
text(-0.75, c(69:64),cex = 1.50 , pos = 2, font = 0.5, c("High","Upper-mid","Lower-mid","Low","Increasing","Decreasing"))
text(-0.75, c(60:55),cex = 1.50 , pos = 2, font = 0.5, c("High","Upper-mid","Lower-mid","Low","Increasing","Decreasing"))
text(-0.75, c(51:46),cex = 1.50 , pos = 2, font = 0.5, c("High","Upper-mid","Lower-mid","Low","Increasing","Decreasing"))
text(-0.75, c(42:37),cex = 1.50 , pos = 2, font = 0.5, c("High","Upper-mid","Lower-mid","Low","Increasing","Decreasing"))
text(-0.75, c(33:28),cex = 1.50 , pos = 2, font = 0.5, c("High","Upper-mid","Lower-mid","Low","Increasing","Decreasing"))
text(-0.75, c(24:19),cex = 1.50 , pos = 2, font = 0.5, c("High","Upper-mid","Lower-mid","Low","Increasing","Decreasing"))
text(-0.75, c(15:10),cex = 1.50 , pos = 2, font = 0.5, c("High","Upper-mid","Lower-mid","Low","Increasing","Decreasing"))
text(-0.75, c(6:1),cex = 1.50 , pos = 2, font = 0.5, c("High","Upper-mid","Lower-mid","Low","Increasing","Decreasing"))


dev.off()
# This has many other options, but it is everything done "by hand"
```

```
    #install.packages("dplyr")                        # Install dplyr for data2
manipulation
  library("dplyr")                                  # Load dplyr

  # Installing the package
  #install.packages("caTools")    # For Logistic regression
  library(caTools)

  #install.packages('randomForest') # For generating random forest model
  library(randomForest)

  #install.packages('caret')                    # classification and
regression training : The library caret has a function to make prediction.
  library(caret)
  #install.packages('e1071', dependencies=TRUE)

  #install.packages("reprtree")




  data2 =  read.csv("C:/Users/mithi/OneDrive - Western Sydney University/
HILDA20/ml/HILDAminPython.csv", stringsAsFactors=TRUE)


  data2$hgage1<- as.numeric(data2$hgage1)


  data2$gh1<- as.numeric(data2$gh1)

  data2$gh10 <- as.numeric(data2$gh10)


  data2$hhra <- as.numeric(data2$hhra)


  data2$edhigh1 <- as.numeric(data2$edhigh1)

  data2$num_clusters <- as.factor(num_clusters)


  split <- sample.split(data2, SplitRatio = 0.8)




  data2_train <- subset(data2, split == "TRUE")
  data2_test <- subset(data2, split == "FALSE")


  data2$num_clusters <- as.factor(data2$num_clusters)

  data2_train$num_clusters <- as.factor(data2_train$num_clusters)
```

```
  #bestmtry <- tuneRF(data2_train,data2_train$num_clusters,stepFactor = 1.2,
improve = 0.01, trace=T, plot= T)

  preds<-data2_train

  preds$num_clusters <- NULL



  mtry <- tuneRF(preds,data2_train$num_clusters)

  best.m <- mtry[mtry[, 2] == min(mtry[, 2]), 1]

  print(mtry)
  print(best.m)


  model <- randomForest(num_clusters~.,data=
data2_train,mtry=best.m,importance=TRUE)

  print(model)


  importance(model)
  TiffFile="C:/Users/mithi/OneDrive - Western Sydney University/HILDA20/
Results/MeanDecreaseAccuracy.tiff"
  tiff(TiffFile, units="in", width=12, height=10, res=300)
  varImpPlot(model)
  dev.off()

  pred_test <- predict(model, newdata = data2_test, type= "class")

  pred_test

  confusionMatrix(table(pred_test,data2_test$num_clusters))
```
